# Albumin Nanoparticle Endocytosing Subset of Neutrophils for Precision Therapeutic Targeting of Inflammatory Tissue Injury

**DOI:** 10.1021/acsnano.1c09762

**Published:** 2022-03-01

**Authors:** Kurt Bachmaier, Andrew Stuart, Abhalaxmi Singh, Amitabha Mukhopadhyay, Sreeparna Chakraborty, Zhigang Hong, Li Wang, Yoshikazu Tsukasaki, Mark Maienschein-Cline, Balaji B. Ganesh, Prasad Kanteti, Jalees Rehman, Asrar B. Malik

**Affiliations:** † Department of Pharmacology and Regenerative Medicine and the Center for Lung and Vascular Biology, The University of Illinois College of Medicine, E403, 835 South Wolcott Avenue, Chicago, Illinois 60612, United States; ‡ Nano Biotherapeutics, Inc., 2201 West Campbell Park Drive, Chicago, Illinois 60612, United States; § Research Resources Center, University of Illinois at Chicago, Chicago, Illinois 60612, United States; ∥ Division of Cardiology, Department of Medicine, The University of Illinois College of Medicine, Chicago, Illinois 60612, United States

**Keywords:** inflammation, drug carriers, nanotechnology, nanotherapeutics, chemokine receptors, neutrophil heterogeneity, bacterial infection

## Abstract

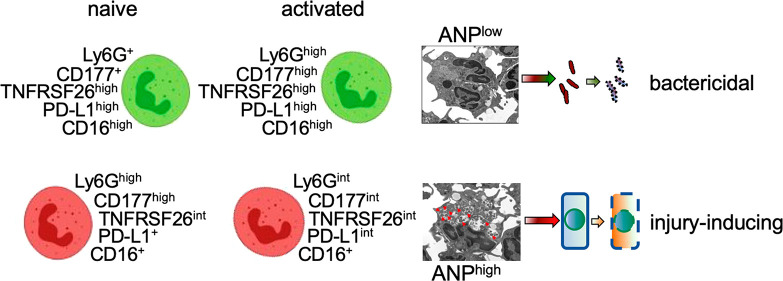

The complex involvement of neutrophils in inflammatory diseases makes them intriguing but challenging targets for therapeutic intervention. Here, we tested the hypothesis that varying endocytosis capacities would delineate functionally distinct neutrophil subpopulations that could be specifically targeted for therapeutic purposes. By using uniformly sized (∼120 nm in diameter) albumin nanoparticles (ANP) to characterize mouse neutrophils *in vivo*, we found two subsets of neutrophils, one that readily endocytosed ANP (ANP^high^ neutrophils) and another that failed to endocytose ANP (ANP^low^ population). These ANP^high^ and ANP^low^ subsets existed side by side simultaneously in bone marrow, peripheral blood, spleen, and lungs, both under basal conditions and after inflammatory challenge. Human peripheral blood neutrophils showed a similar duality. ANP^high^ and ANP^low^ neutrophils had distinct cell surface marker expression and transcriptomic profiles, both in naive mice and in mice after endotoxemic challenge. ANP^high^ and ANP^low^ neutrophils were functionally distinct in their capacities to kill bacteria and to produce inflammatory mediators. ANP^high^ neutrophils produced inordinate amounts of reactive oxygen species and inflammatory chemokines and cytokines. Targeting this subset with ANP loaded with the drug piceatannol, a spleen tyrosine kinase (Syk) inhibitor, mitigated the effects of polymicrobial sepsis by reducing tissue inflammation while fully preserving neutrophilic host-defense function.

Neutrophils as host-defense cells function to maintain tissue hemostasis and to induce sterile inflammatory injury.^1^ They are essential to control microbial infection and can also cause inflammatory tissue damage.^2,3^^4^ Neutrophils, like other myeloid cells, have the ability to readily adapt to signals in their microenvironment.^5−8^ Despite their short half-life in circulation and tissues,^9,10^ neutrophils exhibit transcriptomic, phenotypic, and functional adaptations to environmental cues.^4,11^ Neutrophils adapt to tumor microenvironments by altering transcriptional and functional profiles to either promote or impede tumor growth and metastasis.^12,13^ Similar neutrophil adaptation has been observed in the context of sterile injury and repair,^1,3,14^ allergic asthma,^15^ autoimmune disease,^16^ stroke,^17^ and bacterial infection.^18,19^ Immunomodulatory neutrophils capable of suppressing human T cell proliferation have been identified.^20^ Additionally, the neutrophilic subset of myeloid-derived suppressor cells has emerged in the regulation of immune responses such as in the setting of malignancies.^21,22^ Moreover, as in the case of periodontal neutrophils, interaction with the same microenvironment of commensal biofilms can give rise to neutrophil subsets with distinct phenotypes.^23^ This complex involvement of neutrophils in inflammatory diseases and tissue makes them intriguing but challenging targets for precise therapeutic intervention. Current anti-inflammatory approaches rely on drugs that either do not affect neutrophil function or that may even affect neutrophil activities in a potentially harmful manner.^24^

Unfortunately, there is no single unifying concept to integrate this neutrophilic diversity. The characterization of heterogeneous neutrophil populations by cell surface marker expression has proven difficult,^25^ highlighting the need for new and better means of characterization of neutrophil subsets. Here we show that nanotherapeutics such as nanoparticles made from albumin have the potential to serve as tools for *in vivo* analysis and characterization of the cells that endocytose them.^26^ We surmised that specially formulated albumin nanoparticles (ANP)^27^ could be used experimentally to test the hypothesis that (1) neutrophil subsets adapt to environmental cues and niches in their own subset-specific way, (2) varying endocytosis capacities would delineate functionally distinct neutrophil subpopulations, and (3) ANP could be used for precision drug delivery.

Here, we report that the ability to endocytose ANP defines a neutrophil subset present in bone marrow, blood, spleen, and lung, both under basal conditions and after an inflammatory challenge. By leveraging ANP endocytosis for a molecular characterization of neutrophil subsets, we established signature functional and phenotypic profiles of the neutrophil subset. Furthermore, subset-specific therapeutic targeting proved to be highly effective in ameliorating inflammatory tissue injury.

## Results and Discussion

### Heterogeneity of Albumin Nanoparticle Endocytosis by Mouse Neutrophils

**Figure 1:**
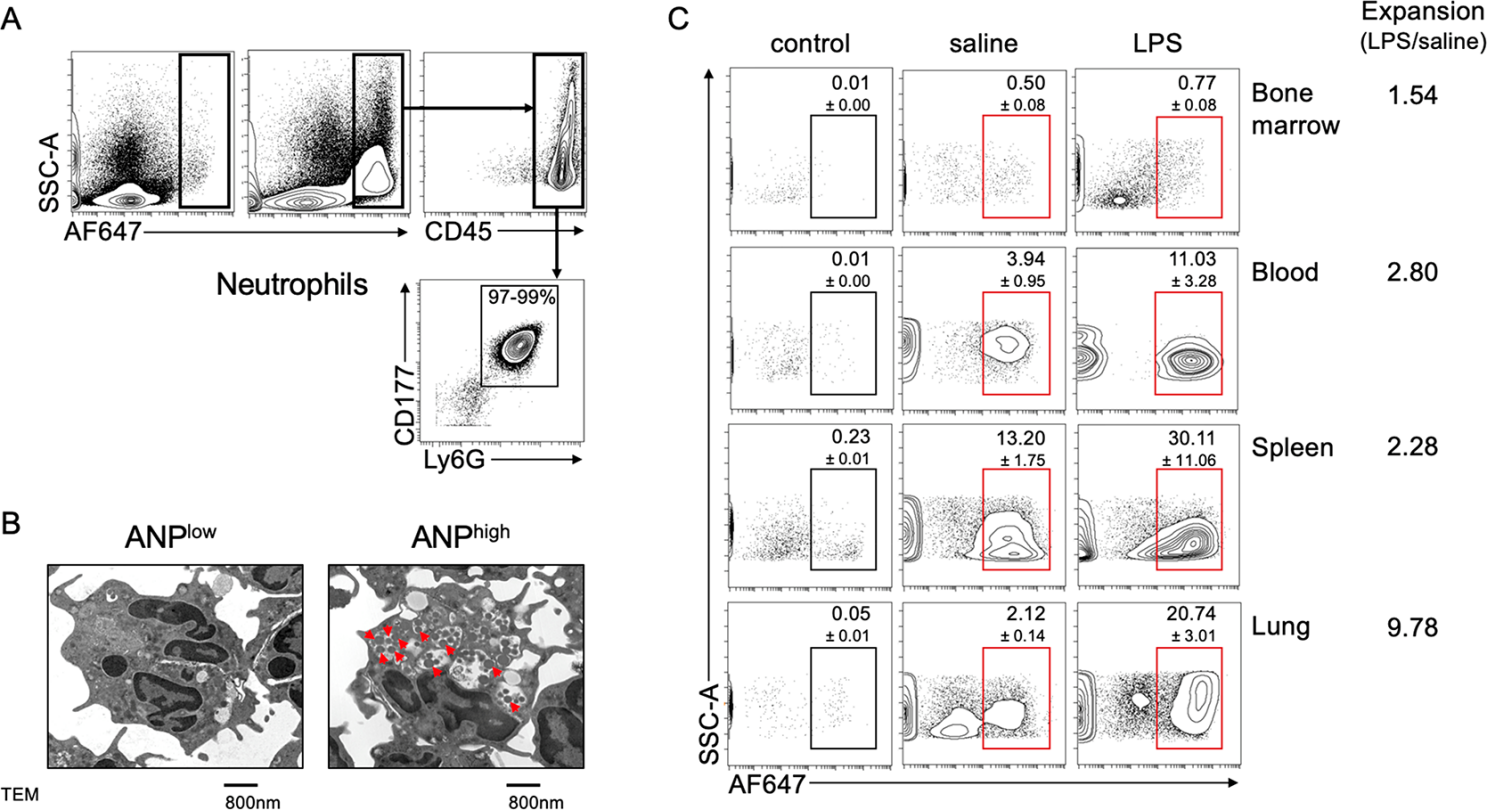
Differential endocytosis of albumin nanoparticles by PMN. (A) Flow cytometric analysis of mouse lung single cell suspensions. Intravenous injection of albumin nanoparticles (ANP) led to endocytosis of ANP by CD177^+^Ly6G^+^ lung PMN. (B) Transmission electron microscopy (TEM) of lung neutrophil that did not endocytose ANP (ANP^low^) and of lung neutrophil that endocytosed ANP (ANP^high^). Red arrows indicate ANP in organelles. (C) Flow cytometric analysis of ANP endocytosis by mouse Ly6G^+^ PMN from various tissues. ANP^high^ PMN were found in bone marrow, peripheral blood, spleen, and lungs at low percentage when compared to PMN that did not endocytose ANP (ANP^low^). Systemic challenge (i.v.) with LPS induced the expansion of ANP^high^ PMN relative to ANP^low^ PMN, which was most pronounced in lungs (ratio of 9.78). Mice (*n* = 4 per cohort) were injected with LPS 6 h prior to euthanasia and with unlabeled ANP (control) 30 min prior to euthanasia; with LPS or saline 6 h prior to euthanasia and with AF647-fluorochrome labeled ANP 30 min prior to euthanasia.

To characterize endocytosis of ANP, we injected fluorescence-labeled ANP intravenously (i.v.) into mice challenged with the lipopolysaccharide of Gram-negative bacteria (2 mg LPS/kg intraperitoneally, i.p.). Thirty minutes after injection, we detected ANP-specific fluorescence in single cell preparations from lungs in CD45^+^ leukocytes but not in CD45^–^ parenchymal cells (Figure 1A). ANP-specific fluorescence was almost exclusively apparent in CD177^+^Ly6G^+^ polymorphonuclear neutrophils (PMN) (Figure 1A). Transmission electron microscopy (TEM) showed two subsets of lung PMN, one that endocytosed a large number of ANP (ANP^high^) and a second that endocytosed few or no ANP (ANP^low^) (Figure 1B). Moreover, a small percentage of PMN from bone marrow, peripheral blood, lungs, and spleens endocytosed i.v.-injected ANP in naive condition (Figure 1C).

Intraperitoneal challenge with LPS induced the expansion of ANP^high^ PMN relative to ANP^low^ PMN (Figure 1C). The expansion of ANP^high^ PMN was most pronounced in the lungs (Figure 1C). These results were consistent with our findings on relative ANP biodistribution as determined in various organs by imaging through IVIS (SI Figure 1). In naive mice, ANP accumulated exclusively in livers and spleens (SI Figure 2). Only after the LPS-challenge did ANP appear in the lungs where ANP-specific radiance was most pronounced (SI Figure 2). Pharmacokinetics studies by measuring blood serum ANP concentrations at various times after injection revealed a serum half-life of ∼23 min (SI Figure 3). We could not detect any ANP in blood sera after i.p. administration of ANP (not shown). These data suggested that the vast majority of ANP was endocytosed by PMN within 30 min after injection and demonstrated that PMN can be separated on the basis of their heterogeneous endocytosis of ANP and that ANP^high^ and ANP^low^ PMN existed simultaneously in vivo in both naive and endotoxemic mice. To test whether human PMN showed similar duality of ANP-endocytosis, we incubated human peripheral blood obtained by venipuncture from healthy volunteers with ANP and found that 2% of CD66b^+^CD10^+^ peripheral blood PMN endocytosed ANP with the percentage increasing to 7% after in vitro stimulation of blood cells with LPS (SI Figure 4). These data indicate that mouse heterogeneity of albumin nanoparticle endocytosis is recapitulated in human PMN.

### ANP Endocytosis Reveals Neutrophil Subset-Specific Transcriptomic Profile

**Figure 2:**
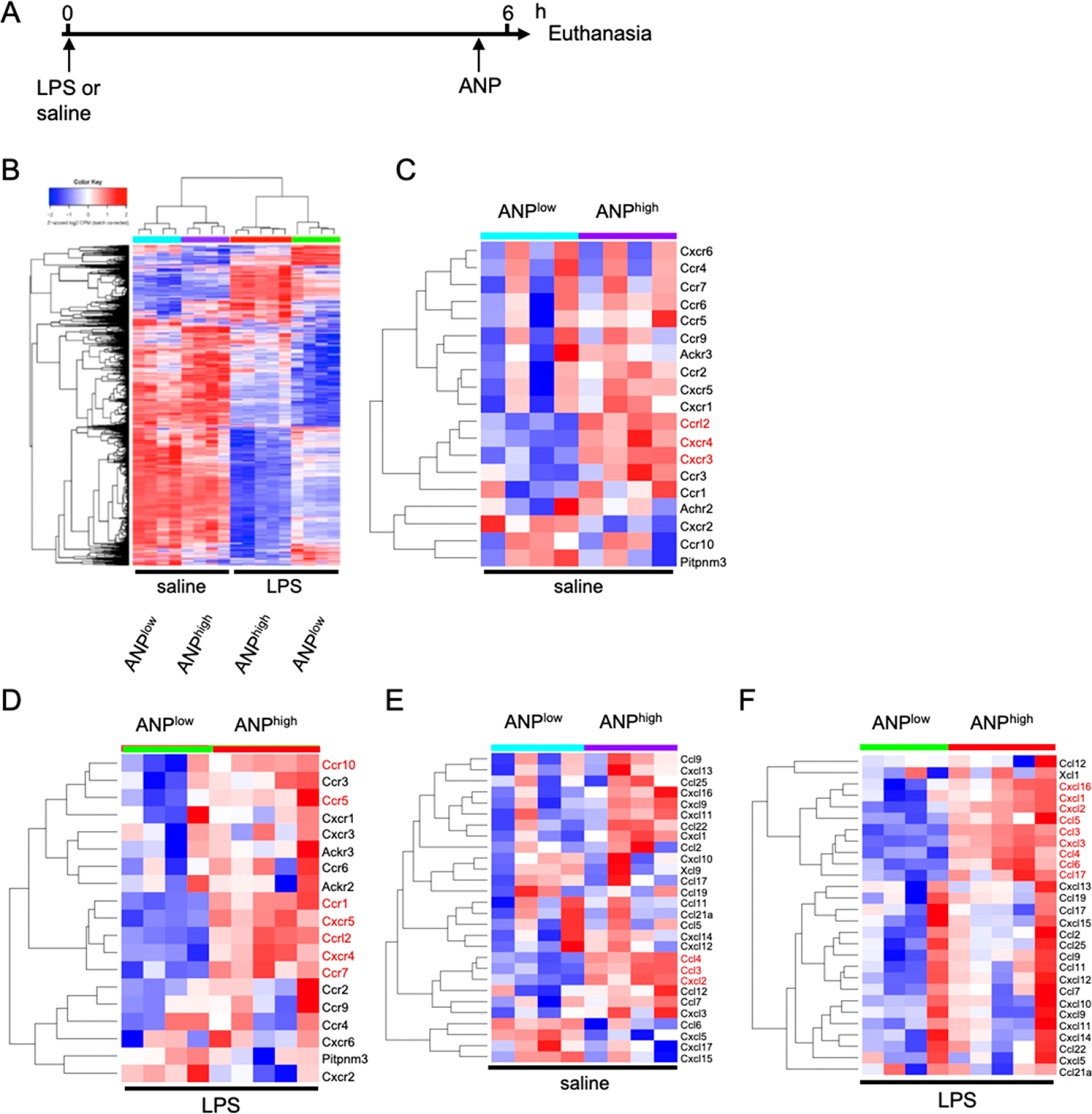
Transcriptomic heterogeneity of lung PMN. Transcriptomic profile of lung ANP^high^ vs ANP^low^ Ly6G^+^ PMN. (A) Mice were challenged for 6 h by i.p. injection of LPS (12 mg/kg) or saline; 5 h 30 min after challenge, mice were injected, i.v., with 1 dose of fluorochrome-labeled ANP, and euthanized 30 min later; Ly6G^+^ PMN from lungs were sorted according to ANP endocytosis and then mRNA was processed for RNA-Seq. (B) Dendrogram and heat map showing normalized gene expression data of biological replicate samples from saline injected controls, ANP^low^ (blue), ANP^high^ (purple), or of LPS-challenged mice, ANP^high^ (red), ANP^low^ (green). (C–F) Heatmaps of chemokine receptors or chemokines. Chemokine receptor expression in lung PMN from saline injected mice (C) or LPS-challenged mice (D). Chemokine expression in PMN from (E) saline injected mice or (F) LPS-challenged mice. Significantly higher expression values are shown in red, lower expression values in blue. Representative data from 3 independent experiments.

We performed an unbiased analysis of lung PMN transcriptomic profiles using RNA-Seq We injected LPS or saline i.v., and to minimize any effect of ANP endocytosis per se on PMN transcriptomic activity, ANP exposure was limited to a 30 min period prior to euthanasia (Figure 2A). We euthanized the mice 6 h after LPS or saline injection and prepared a single cell suspension from lungs, sorted Ly6G^+^ PMN by flow cytometry according to their ANP endocytosis into ANP^low^ and ANP^high^ PMN (Figure 2A). Immediately after sorting, we prepared PMN mRNA for RNA-Seq analysis.

We generated heatmaps and dendrograms to represent the normalized PMN gene expression data (Figure 2B and SI Figure 5). We found that the biological replicates clustered into 4 groups with distinct transcriptomic profiles; i.e., mRNA profiles defined PMN from LPS-challenged or saline-injected control mice were distinct in ANP^low^ and ANP^high^ PMN (Figure 2B). Using MetaCore Pathway analysis to identify pathway differences between ANP^high^ and ANP^low^ PMN, we found that the pathways regulating immune response and immune cell migration were significantly over-represented in ANP^high^ PMN (SI Table 1). Specifically, the pathways containing chemokine receptors were significantly enriched in ANP^high^ PMN, suggesting that this PMN subset has the capacity of higher trafficking and migration, consistent with the increased fraction of ANP^high^ PMN we observed in lungs (see Figure 1). Consistently, we found that chemokine receptors were over-represented 6.5-fold (*p* = 0.01, Fisher’s Exact test) in ANP^high^ PMN derived from LPS-challenged mice and 4-fold in ANP^high^ PMN of naive mice (*p* = 0.0005, Fisher’s Exact test). Moreover, chemokines were overrepresented 3.9-fold (*p* = 0.04, Fisher’s Exact test) in ANP^high^ PMN of LPS-challenged mice and 3-fold in ANP^high^ PMN of naive mice (*p* = 0.0013, Fisher’s Exact test).

To identify the chemokine receptors for each PMN subset, we generated separate heatmaps for chemokine receptors, plotting all genes with CPM > 0.25 (10 reads at sequencing depth of 40 M reads) regardless of differential expression levels. In naive mice, ANP^high^ PMN showed relative overexpression of the chemokine receptors Cxcr3, Cxcr4, and Ccrl2 (Figure 2C). In LPS-challenged mice ANP^high^ PMN showed relative overexpression of chemokine receptors Ccr1, Ccr5, Ccr7, Ccr10, Ccrl2, Cxcr4, and Cxcr5 (Figure 2D). We next assessed the expression of chemokines in ANP^high^ PMN and ANP^low^ PMN. In saline-injected control mice, ANP^high^ PMN were significantly enriched for the expression of chemokines Ccl3, Ccl4, and Cxcl2 (Figure 2E). In LPS-challenged mice, ANP^high^ PMN demonstrated relative overexpression of the chemokines Ccl3, Ccl4, Ccl5, Ccl6, Ccl17, Cxcl1, Cxcl2, Cxcl3, and Cxcl16 (Figure 2F). These data suggested that ANP^high^ PMN were capable of releasing inordinate amounts of chemokines and that ANP^high^ PMN would respond more readily to chemokine gradients than ANP^low^ PMN.

**Figure 3:**
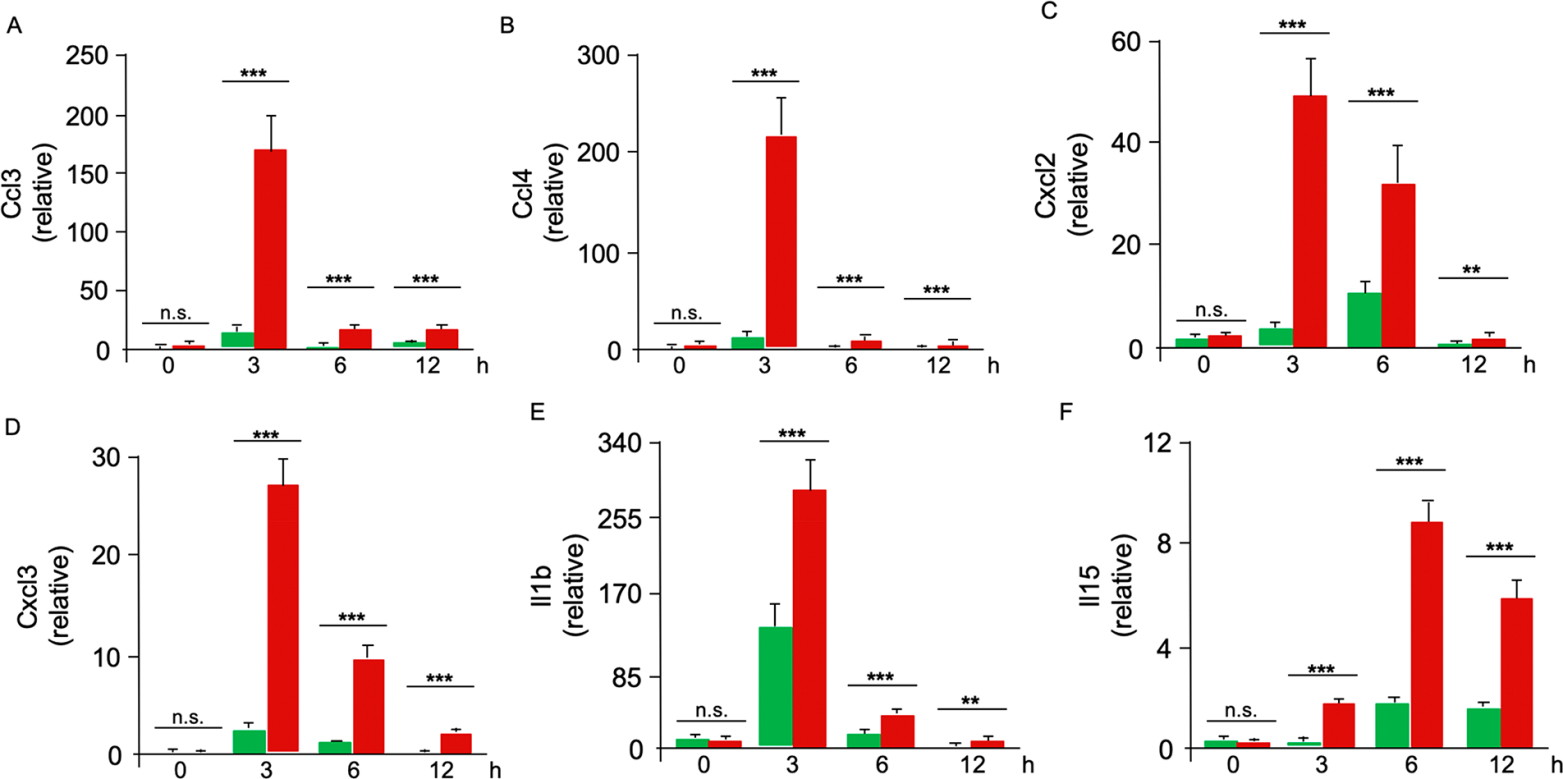
Kinetics of chemokine and cytokine mRNA expression in lung PMN subsets. Mice were treated with either saline (0 h) or LPS for 3, 6, and 12 h. Fluorochrome labeled ANP were injected 30 min before euthanasia. Ly6G^+^ PMN were sorted into ANP^low^ and ANP^high^. qPCR analysis of ANP^low^ (green columns) or ANP^high^ (red columns) PMN. qPCR analysis of (A) Ccl3; (B) Ccl4; (C) Cxcl2; (D) Cxcl3; (E) Il1b; (F) Il15. Representative data from 3 independent experiments. Mean values and SD. n.s., not significant. ** *p* < 0.01; *** *p* < 0.001 (Student’s *t* test).

Based on our RNA-Seq data and MetaCore Pathway analysis, we selected chemokines and cytokines over-represented in ANP^high^ PMN to validate their expression levels by qPCR and to determine the kinetics of their expression in response to LPS-stimulation. We found that mRNA expression levels of Ccl3, Ccl4, Cxcl2, and Cxcl3 (Figure 3A–D) were significantly greater in ANP^high^ PMN than ANP^low^ PMN at 3, 6, and 12 h after in vivo LPS challenge. Ccl3 (Figure 3A) and Ccl4 (Figure 3B) expression, in particular, was much greater in ANP^high^ than ANP^low^ PMN. These results suggested that ANP^high^ PMN are specialized inflammatory cells, which could contribute to the recruitment and activation of additional myeloid cells via the release of chemokines.^28^

We next measured cytokines known to control inflammatory responses, i.e., IL-1β and IL-15.^29^ The cytokine IL-1β, which is essential for host-defense function and amplification of inflammation, was induced ∼21-fold in ANP^low^ PMN and ∼78-fold in ANP^high^ PMN after LPS challenge (Figure 3E), revealing distinct responses to equal stimuli by the two subsets of PMN. Similarly, expression of the pleiotropic cytokine IL-15 was also significantly greater in ANP^high^ than ANP^low^ PMN in lungs 3, 6, and 12 h after LPS challenge (Figure 3F).

### High-Dimensional Analysis of Cell Surface Marker Expression Confirms Neutrophil Heterogeneity

**Figure 4:**
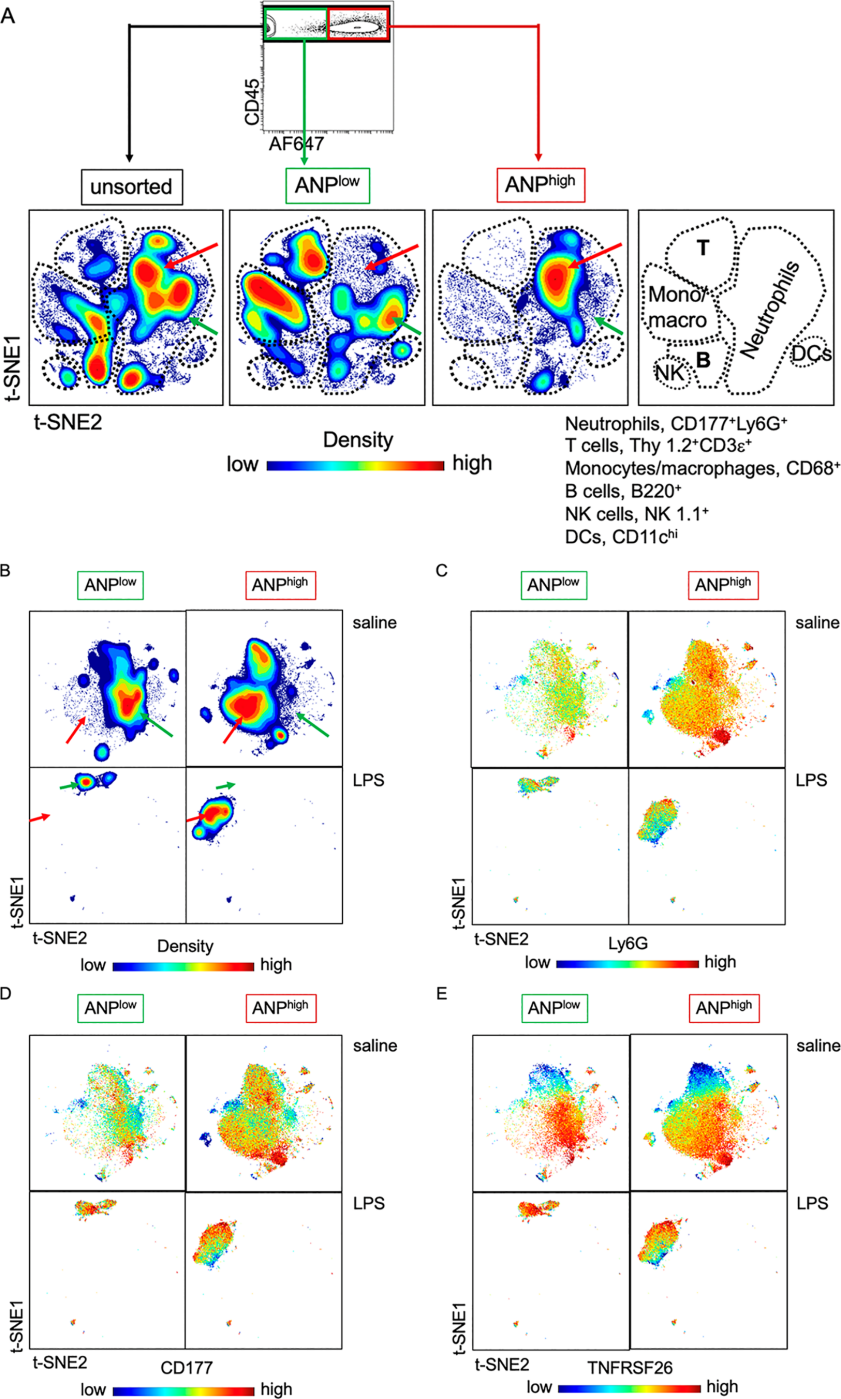

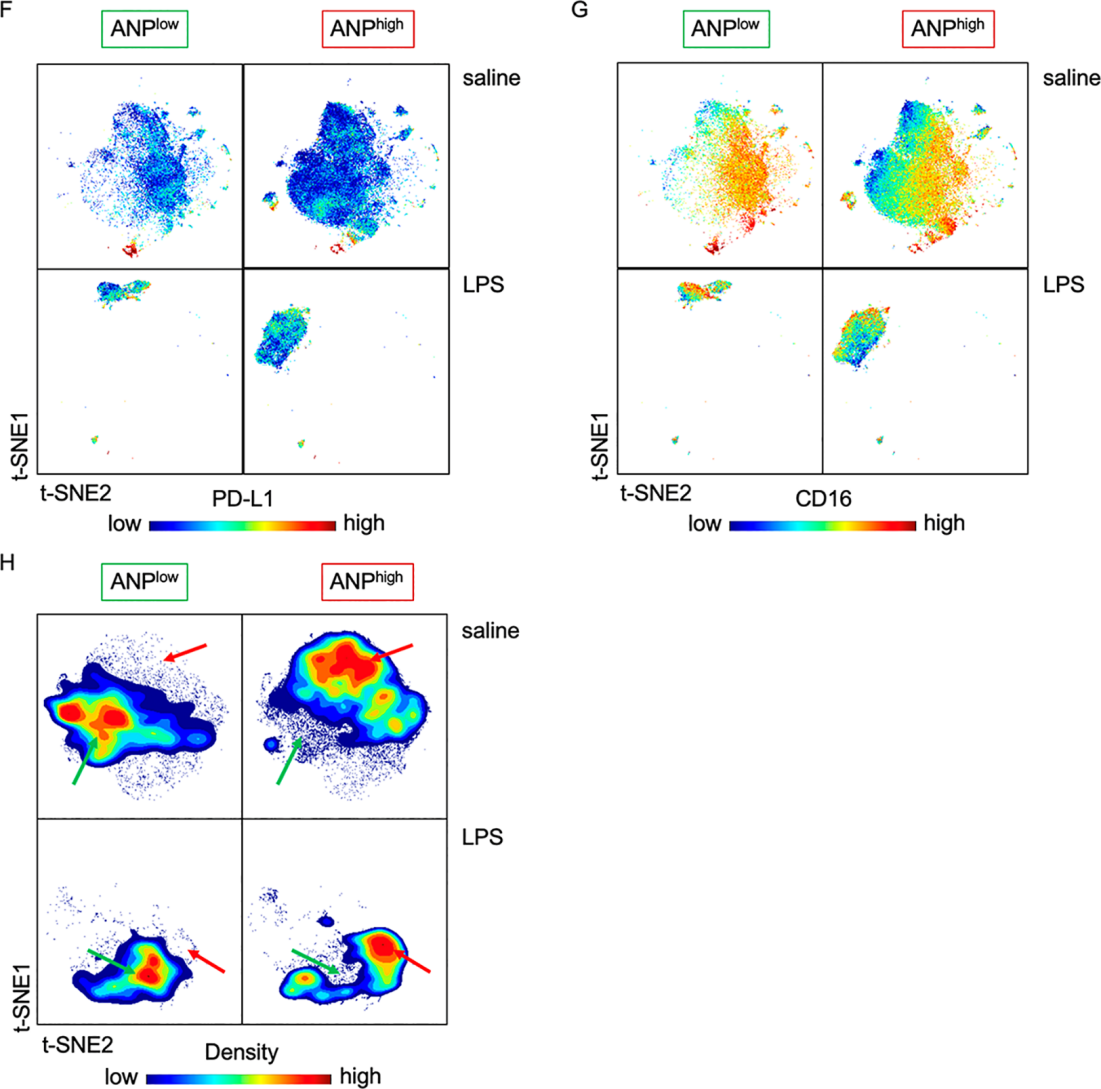
PMN subset cell-surface-marker expression. (A) Contour plots according to cell density of lung CD45^+^ leukocytes. The three maps were generated by considering 38 cell surface markers (encoding genes listed in Table 1). Unsupervised grouping of individual cells. viSNE visualization of high dimensional single-cell data separated most major leukocyte subtypes (shown in right-hand panel). Unsupervised grouping was confirmed by the markers listed for PMN, T cell B cells natural killer (NK) cells, and dendritic cells (DCs). The contours in each plot (representing cell density) delineated cell heterogeneity. Arrows indicate differences in abundance within the neutrophil compartment. Green arrows indicate ANP^low^ and red arrows ANP^high^ PMN. Mice (*n* = 4 per cohort) were injected with LPS 6 h prior to euthanasia and with AF647-fluorochrome labeled ANP 30 min prior to euthanasia. Lung CD45^+^ leukocytes were left unsorted or sorted by flow cytometry according to their ability to endocytose ANP into ANP^high^ and ANP^low^ cells. Cells were then subjected to mass cytometry (CyTOF), and visualized by viSNE, based on the t-Distributed Stochastic Neighbor Embedding (t-SNE) algorithm. (B) Contour plots according to cell density of lung CD177^+^Ly6G^+^ PMN generated by considering 38 cell surface markers (encoding genes listed in Table 1) from mice injected with saline or LPS. The viSNE maps were generated by unsupervised grouping of individual cells. Arrows indicate differences in abundance between neutrophil subsets. Green arrows, ANP^low^; red arrows ANP^high^. (C–E) Spectrum colored dot plots. Intensities of protein expression of markers are shown on viSNE map as spectrum colored dots (low in blue, high in red). (F, G) Spectrum colored dot plots. Intensities of protein expression of markers are shown on viSNE map as spectrum colored dots (low in blue, high in red). (H) Contour plots according to cell density of lung CD177^+^Ly6G^+^ PMN generated by considering 5 cell surface markers (antigens listed in Table 2) from mice injected with saline or LPS. The 5 viSNE maps were generated by unsupervised grouping of individual cells. Arrows indicate differences in abundance between neutrophil subsets. Green arrows, ANP^low^; red arrows ANP^high^.

To validate heterogeneity at the single cell and protein expression level, we chose markers present at the cell surface based on their different mRNA expression in ANP^high^ and ANP^low^ lung PMN (see Figure 2) for high-dimensional analysis using mass cytometry (CyTOF). Our inclusion criteria were *q*-values of less than 0.005 (FDR lower than 0.5%) and log_2_-fold changes of greater or less than 0.6 (Table 1). We also included canonical markers of PMN (CD11b, Ly6G, CD177, CXCR2, CXCR4), natural killer cells (CD16, NK1.1), T lymphocytes (Thy 1.2, CD3ε), B lymphocytes (B220), dendritic cells (CD11c), and monocytes and macrophages (CD68) (Table 1). We stained with elemental isotope-conjugated antibodies specific for 38 surface markers (Table 1). We then analyzed the expression of these markers simultaneously on individual lung CD45^+^ leukocytes using viSNE, based on the t-Distributed Stochastic Neighbor Embedding (t-SNE) algorithm.^30^ The t-SNE projection of cell clusters showed clearly separated cell subsets in space, accurately distinguishing T and B lymphocytes, monocytes/macrophages, natural killer (NK) cells, and dendritic cells (Figure 4A). t-SNE analysis confirmed (see Figure 1) that ANP were mostly endocytosed by PMN (Figure 4A). Importantly, the distributions of cell surface marker expression generated contour-plot maps that were distinct for ANP^high^ and ANP^low^ lung CD177^+^Ly6G^+^ on PMN from both naive and LPS-challenged mice (Figure 4B). Variation in the expression of individual cell-surface markers became apparent in viSNE dot blots (Figure 4C–G). We then reduced the number of simultaneously measured cell surface receptors. We compiled a list of receptors on the basis of their different cell surface expression (median intensity values in CyTOF) on ANP^low^ and ANP^high^ lung PMN. Five markers that were distinct between ANP^low^ and ANP^high^ lung PMN (Table 2) were sufficient to delineate ANP^high^ and ANP^low^ PMN in viSNE-generated contour plots (Figure 4H) and provided a signature marker profile to distinguish neutrophil subsets. We next investigated whether ANP^high^ were indeed functionally different from ANP^low^ PMN.

**Table 1:** Cell Surface Markers Differentially Expressed in ANP^high^ and ANP^low^ PMN^a^

	saline	LPS
Gene	logFC (ANP^low^/ANP^high^)	logFC (ANP^low^/ANP^high^)
Tnfrsf26	–1.73262421	–2.657676
Clec7a	–1.044724054	–2.078300
Cd244	–0.816499782	–1.970690
Cxcr3	–1.621891364	–1.621890
Tnfrsf8	–0.963506698	–1.577790
Il15ra	–0.048885223	–1.420680
Pecam1	–0.272222647	–1.358090
Cd274 (PD-L1)	–1.328938411	–1.357850
Tlr6	–0.805369715	–1.287680
Cd68	–0.878052769	–1.211180
Tnfrsf9	–0.498199032	–1.205280
Procr (CD201)	–0.099146427	–1.199040
Cd40	–0.544657965	–1.161850
**Cxcr4**	–0.919071003	–1.158270
Tlr1	–0.37362063	–1.116150
Ccrl2	–1.527591012	–1.089050
**Cd3e**	–0.069573722	–0.978163
Cd74	–0.608901968	–0.958716
Jaml	0.026618296	–0.945136
**Itgax** (CD11c)	–0.800173707	–0.928278
Cd84	–0.193804427	–0.846793
Cd115	–0.505175069	–0.763241
Ccr1	–0.232941656	–0.732656
**Klrb1** (NK1.1)	0.492338778	–0.682963
Itgb7	–0.417413035	–0.649137
Ccr7	–0.24938707	–0.629915
**Sell** (CD62L)	0.119925807	–0.529186
Icam1	–1.075309125	–0.314039
**Thy1**	–0.095529324	–0.271720
**Ptprc** (B220)	–0.458431681	–0.156455
Fcgr3 (CD16)	0.257091771	–0.119756
**Cxcr2**	0.555029693	0.449032
**Itgam** (CD11b)	0.298204347	0.693555
**Ly6g**	1.884798818	1.081058
**Cd177**	1.610499768	1.148760
Cd81	0.110210089	1.395810
Nt5e (CD73)	1.175626193	1.436910
Cd55	0.840994987	1.656220

a logFC (ANP^low^/ANP^high^) of mRNA expression of 38 genes encoding cell surface markers. Canonical marker of leukocyte subtypes in bold. Names of recognized epitopes in brackets. Mice were injected with LPS or saline 6 h prior to euthanasia and with AF647-fluorochrome labeled ANP 30 min prior to euthanasia. Lung Ly6G^+^ PMN were sorted according to ANP-endocytosis and their mRNA was processed for RNA-Seq.

**Table 2:** Signature Cell Surface Receptor Profile of Neutrophil Subsets^a^

	saline	LPS
antigen	arcsinh ratio (ANP^low^/ANP^high^)	arcsinh ratio (ANP^low^/ANP^high^)
Ly6G	0.43655	–0.10201
CD177	0.33167	–0.28025
TNFRSF26	–0.29305	–0.29533
PD-L1	–0.36241	–0.18730
CD16	–0.57493	–0.35156

a Arcsinh ratio (ANP^low^/ANP^high^) of median intensity values of lung CD177^+^Ly6G^+^ PMN. Mice (*n* = 4 per cohort) were injected with LPS or saline 6 h prior to euthanasia and with AF647-fluorochrome labeled ANP 30 min prior to euthanasia. Lung CD45^+^ leukocytes or sorted by flow cytometry according to their ability to endocytose ANP into ANP^high^ and ANP^low^ cells. Cells were then subjected to mass cytometry (CyTOF).

### ANP^high^ and ANP^low^ Neutrophils Are Functionally Distinct

**Figure 5:**
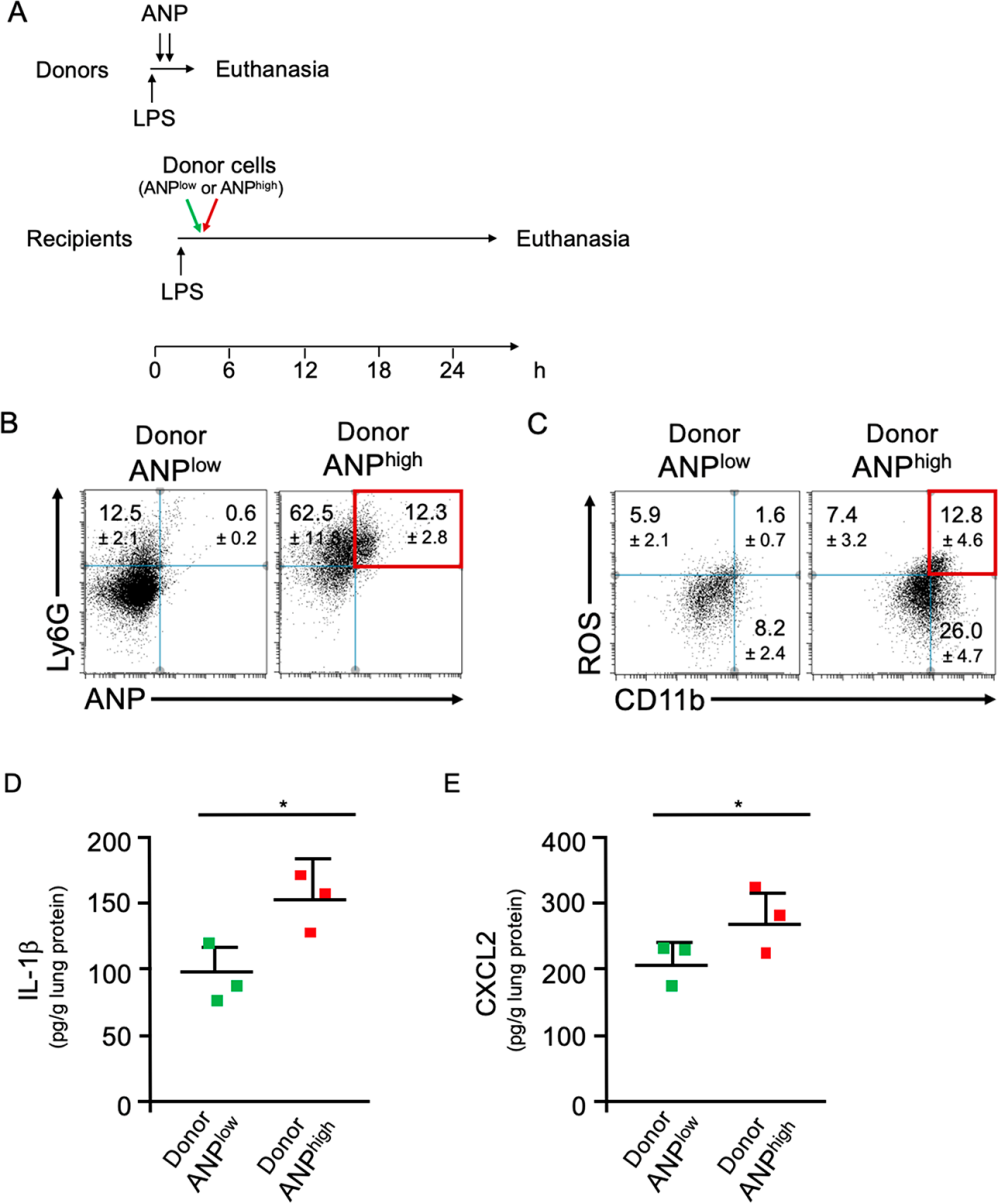
ANP^high^ PMN transfer inflammation. (A) Timeline of adoptive transfer. Donor mice were challenged with a lethal dose of LPS and injected with two doses of ANP labeled with the stable fluorochrome AF647. ANP^high^ PMN (8 × 10^5^) or, as controls, an equal number of ANP^low^ lung Ly6G^+^ PMN were adoptively transferred by i.v. injection into syngeneic recipient mice. (B) Flow cytometric analysis of lung cells from mice that received ANP^low^ or ANP^high^ donor cells. Dot blot. Percentages of Ly6G^+^ANP^+^ cells (red) were significantly greater in mice that received ANP^high^ donor cells as compared to mice that received ANP^low^ donor cells. *p* < 0.001 (Student’s *t* test). (C) Flow cytometric analysis of lung cells from mice that received ANP^low^ or ANP^high^ donor cells. Dot blot. Percentages of ROS^+^CD11b^+^ (red) were significantly greater in mice that received ANP^high^ donor cells as compared to mice that received ANP^low^ donor cells. (D) Concentrations of IL-1β in lung tissue extracts from mice that have received ANP^low^ or ANP^high^ donor cells. (E) Concentrations of CXCL2 in lung tissue extracts from mice that have received ANP^low^ or ANP^high^ donor cells. Squares represent values from individual mice and lines indicate mean values + SD *p < 0.05 (Student’s *t* test). Representative data from 3 independent experiments are shown.

ANP^high^ PMN displayed a distinct proinflammatory profile when compared to ANP^low^ PMN from the same mice. We therefore determined whether adoptively transferring ANP^high^ PMN from donors into syngeneic recipient mice would induce lung inflammation in recipient mice. To induce the sequestration of optimally activated neutrophils in the lungs of donor mice, donor BALB/c mice were challenged with a lethal dose of LPS [30 mg/kg] and injected with fluorochrome-labeled ANP. ANP^high^ PMN (8 × 10^5^) or, as controls, with an equal number of ANP^low^ PMN from the lungs of the same donors were injected i.v. into recipient mice that had been pretreated (2 h prior to adoptive transfer) with a sublethal dose of LPS [1 mg/kg, i.p.] a dose sufficient to activate their endothelium, a prerequisite for initiating neutrophilic lung inflammation.^31^ At 24 h after adoptive transfer, we assessed lung inflammation in recipient mice (Figure 5A). We found ANP^+^Ly6G^+^ PMN in lungs of recipient mice, indicating donor cell homing to lungs of recipient mice (Figure 5B). Transfer of donor ANP^high^ PMN significantly increased neutrophilic lung inflammation in the recipient mice when compared to mice receiving donor ANP^low^ cells (Figure 5B). Moreover, there were significantly more Ly6G^+^ROS^+^ PMN after transfer of ANP^high^ PMN, when compared to control transferred ANP^low^ PMN (Figure 5C). The concentration of the inflammatory cytokine IL-1β was also significantly greater in lung tissue lysates of mice receiving ANP^high^ than those receiving ANP^low^ PMN (Figure 5D). Additionally, the concentration of the inflammatory chemokine CXCL2 was increased in lung lysates of mice receiving ANP^high^ than in those receiving ANP^low^ PMN (Figure 5E). These data demonstrated the intrinsic ability of ANP^high^ PMN to transfer lung inflammation.

**Figure 6:**
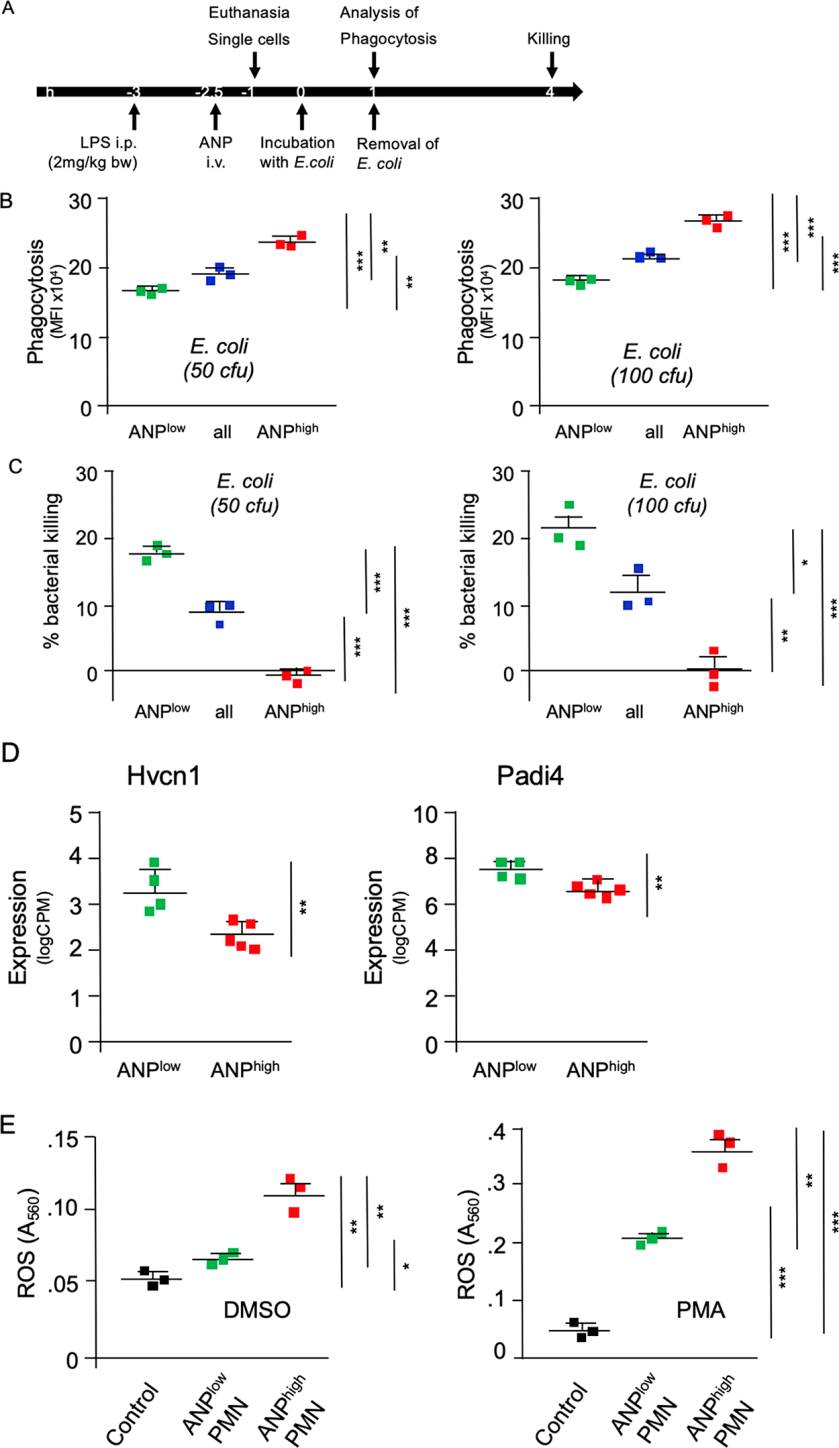
Functional heterogeneity of lung PMN. (A) Timeline of assays. (B) *E. coli* phagocytosis. PMN were incubated with *E. coli* bacteria (50 cfu or 100 cfu) for 1 h. *E. coli*-specific fluorescence is shown. (C) Killing of intracellular *E. coli* bacteria. Single cell suspensions of lung unsorted Ly6G^+^ PMN (blue) or sorted according to endocytosis of ANP (low, green; or high, red). PMN were incubated with *E. coli* bacteria (50 cfu or 100 cfu) for 1 h, and then washed and incubated for additional 3 h to evaluate bacterial killing. *E. coli*-specific fluorescence at 4 h relative to *E. coli*-specific fluorescence at 1 h, corresponding to bacterial killing. Average (*n* = 3) of fluorescence detected at 1 h = 100%; % killing = 100 – percentage of fluorescence detected at 4 h post start of incubation. Markers represent results from individual mice*p < 0.005, **p < 0.002, ***p < 0.0002. (D) mRNA expression of hydrogen voltage gated channel 1 (Hvcn1) and peptidyl arginine deiminase 4 (Padi4) in ANP^low^ and ANP^high^ lung PMN after LPS-challenge. (E) ROS production by ANP^high^ is not impaired. Mice were injected i.p. with LPS and 2 h 30 min later with ANP (i.v.) and euthanized 30 min thereafter. Single cell suspensions of the lungs Ly6G^+^ PMN were sorted according to endocytosis of ANP. Cells were incubated and stimulated with DMSO or phorbol ester PMA. Control, unsorted PMN from naive mice. Squares represent results from individual mice. *p < 0.005, **p < 0.002, ***p < 0.0001.

We next characterized two essential neutrophilic functions, phagocytosis of bacteria and intracellular bacterial killing in ANP^low^ vs ANP^high^ PMN. Endocytosis of ANP is largely facilitated by CD16 (FcγRIII).^27^ Because binding of *E. coli* bacteria to CD16 could trigger a signaling cascade (FcRγ-phosphorylation, recruitment of tyrosine phosphatase SHP-1 and phosphatidylinositide-3 kinase (PI3K) dephosphorylation) that inhibits *E. coli* phagocytosis,^32^ we analyzed whether in vivo administration of ANP interfered with phagocytosis of *E. coli* bacteria (Figure 6A). ANP^high^ PMN that were derived from lungs of mice 3 h after sublethal i.p. LPS-challenge endocytosed *E. coli* bacteria significantly more efficiently than ANP^low^ PMN (derived from the same lungs) (Figure 6B). We found, however, that ANP^low^ PMN eliminated *E. coli* bacteria more efficiently than ANP^high^ PMN (Figure 6C). Furthermore, expression of genes that are essential for intracellular bacterial killing, like hydrogen voltage gated channel 1 (Hvcn1)^33^ and peptidyl arginine deiminase 4 (Padi4),^34^ was significantly higher in ANP^low^ than in ANP^high^ PMN (Figure 6D), a finding consistent with the more efficient killing of intracellular bacteria by ANP^low^ PMN. We next analyzed whether ANP^high^ PMN were compromised in the generation and release of reactive oxygen species (ROS). In an ex vivo assay, ANP^high^ PMN released significantly greater ROS than the ANP^low^ PMN counterparts (Figure 6E), consistent with the differences in antibacterial function of the subsets. We surmised that the fundamental difference in ANP-endocytosis could be exploited for therapeutic purposes to specifically target tissue destructive PMN while not affecting PMN required for antimicrobial function in vivo.

### Treatment Targeted at a Subset of Neutrophils Reduced Inflammation and Injury in Inflammatory Lung Injury (ALI) Models

**Figure 7:**
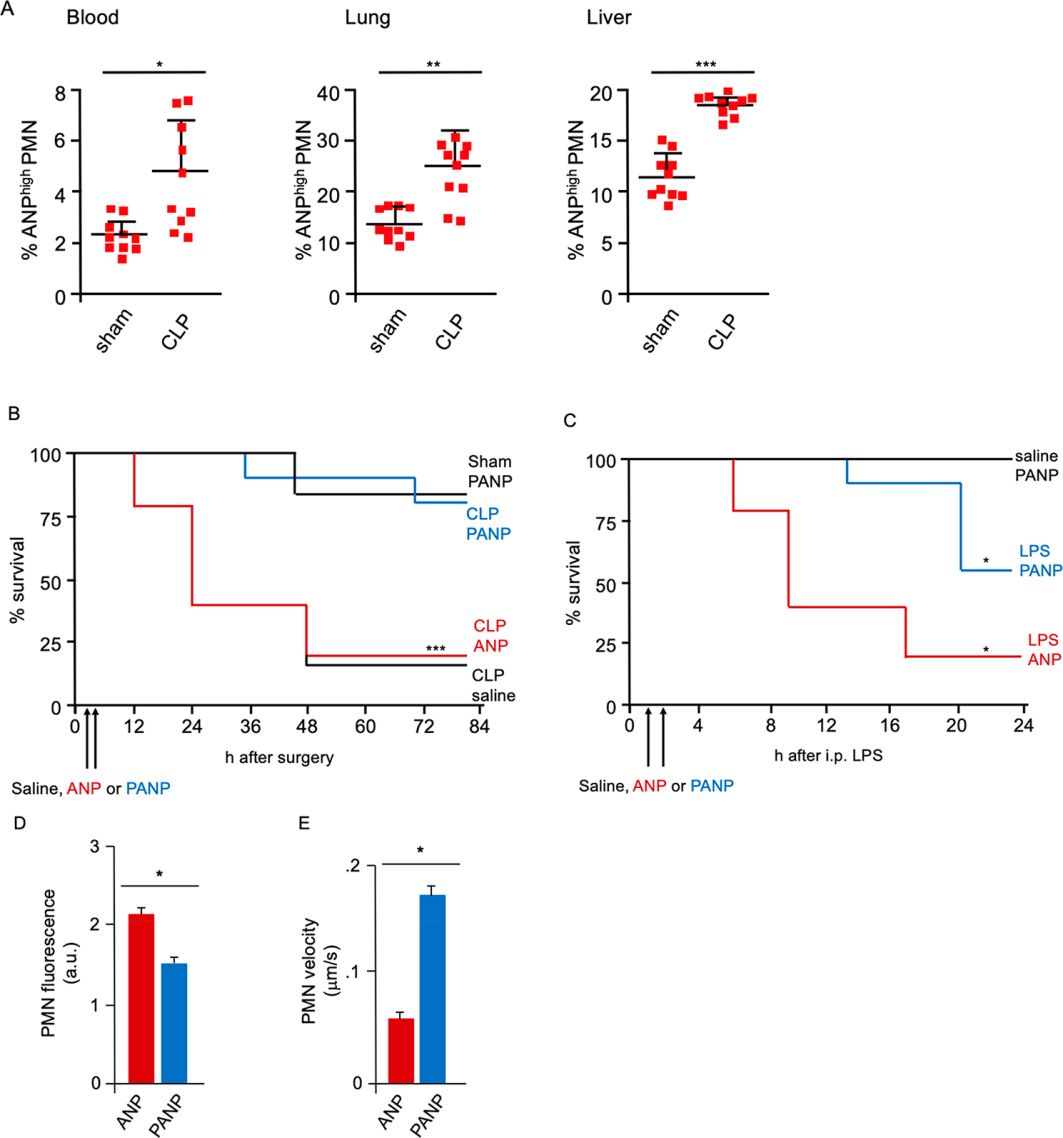
Therapeutic targeting ANP^high^ PMN. (A) Flow cytometric analysis of peripheral blood, lung, or liver Ly6G^+^ PMN. % of cells with high ANP-specific fluorescence 6 h after surgery. Squares represent values from individual mice and lines indicate mean values + SD (B,C) Kaplan–Meier survival curves. (B) i.v. injections of PANP or ANP given 2 and 4 h after CLP. (C) i.v. injections of PANP or ANP given at 1 and 2 h after i.p. challenge with LPS [30 mg/kg]. Representative data 10 mice per treatment group. *p < 0.05, *** *p* < 0.0001. (D,E) Number and velocity of lung Ly6G^+^ PMN determined by two-photon in vivo microscopy of lungs. Velocity in μm/s. i.p. injections of LPS [30 mg/kg], 6 h prior to, and of ANP or PANP 3 and 4 h prior to start of imaging. (D) Fluorescence (a.u.) of Ly6G^+^ PMN. *n* = 5 for ANP, *n* = 6 for PANP. (E) Quantitative analysis of PMN velocity (*n* = 40 for ANP, *n* = 49 for PANP). PMN velocity determined for at least 1 min in the field of view. Error bars indicate SD * *p* < 0.01*, **p < 0.005, *** *p* < 0.001.

At 6 h after cecal ligation and puncture (CLP) or sham operation (laparotomy plus cecal ligation without puncture of the cecum),^35^ and two sequential i.v. injections of ANP, we observed significantly greater expansion of ANP^high^ PMN relative to ANP^low^ PMN in mice subjected to CLP than sham operated controls (Figure 7A). Expansion of ANP^high^ PMN was apparent in peripheral blood, lung, and liver (Figure 7A).

We next tested the hypothesis that changing the function of ANP^high^ PMN by pharmaceutical means could improve the outcome of experimental CLP. We used the drug piceatannol, a Syk inhibitor,^36,37^ that is readily incorporated into ANP due to its poor water solubility, to inhibit Syk-mediated β_2_-integrin-dependent adhesion in ANP^high^ PMN.^27^ We found that therapeutic administration of piceatannol-loaded ANP (PANP) protected mice from lethal polymicrobial sepsis (Figure 7B). Treatment with two i.v. injections of PANP, 2 and 4 h after CLP, reduced lethality to the rate seen in control sham-operated mice (Figure 7B). ANP vehicle treated mice had a lethality rate similar to saline-injected controls (Figure 7B), indicating that ANP by themselves had no discernible effect on neutrophil function in vivo. These data demonstrated that precision targeting the ANP^high^ subset of PMN could reduce CLP-lethality to the level of sham controls. Similarly, in the absence of polymicrobial infection, but after i.p. challenge with a lethal dose of the endotoxin LPS (LD_80_), mice treated with two sequential i.v. injections of PANP at 1 and 2 h after LPS challenge showed significantly reduced mortality when compared to ANP-vehicle treated controls (Figure 7C).

To understand the mechanisms of PANP treatment, we used two-photon microscopy to visualize the effects of PANP-treatment lung PMN in vivo.^38^ When we monitored the behavior of Ly6G^+^ PMN in the lung microvasculature, we found that PMN numbers increased and their velocity decreased significantly in LPS challenged mice (SI video). Treatment of mice with PANP, however, significantly increased the velocity of Ly6G^+^ PMN in the lung microvasculature and reduced the number of Ly6G^+^ PMN as compared to ANP-vehicle treated controls (SI video, Figure 7D,E). These data suggested a mechanism of PANP action that reduced the exposure of lungs to noxious mediators generated by ANP^high^ PMN.

**Figure 8:**
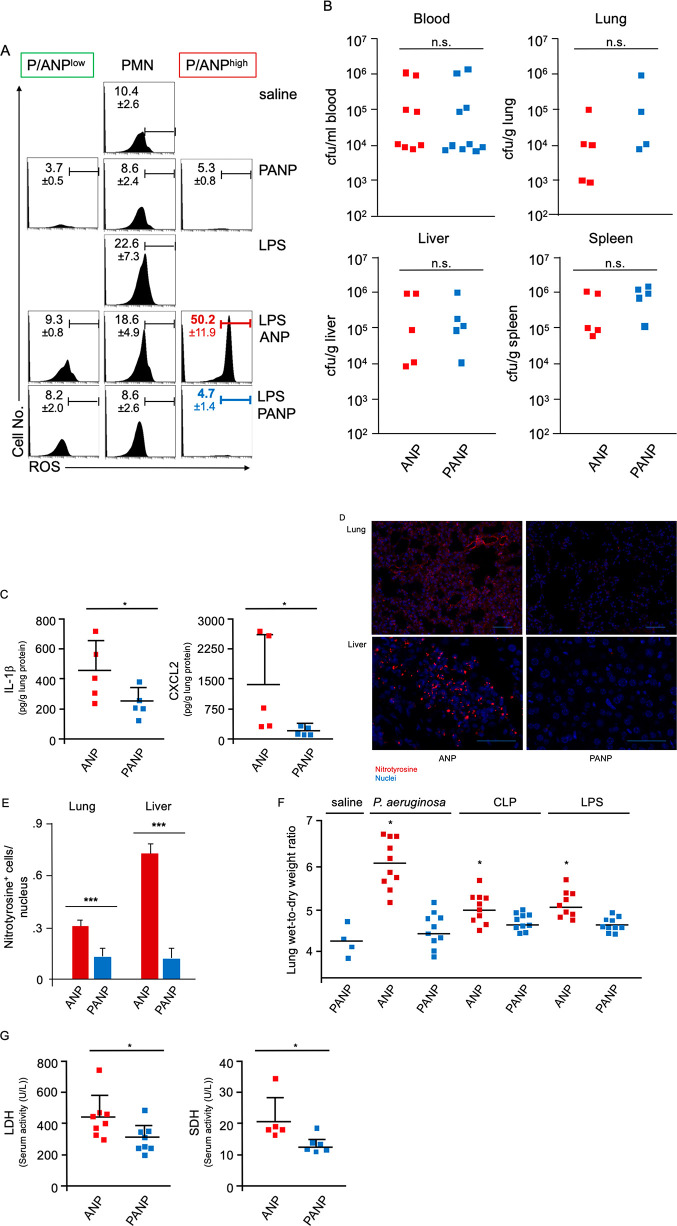
Targeting ANP^high^ PMN improves tolerance of polymicrobial infection. (A) Flow cytometric analysis of intracellular ROS in lung PMN. Mice were treated with 2 consecutive i.v. injections of PANP or ANP 1 and 2 h after i.p. challenge with LPS, and ROS was measured 6 h after challenge. Histograms representing ROS in all Ly6G^+^ PMN, ANP^high^, or PANP^high^ (P/ANP^high^). ROS production was measured using DHR-123. PMN ROS is significantly reduced by PANP treatment (from average 50.2% ROS^+^ cells to 4.7%). Representative data from a minimum of 3 mice per treatment group are shown. (B) Bacterial load (cfu) in peripheral blood, lungs, livers, or spleens of mice post-CLP. PANP treatment, given 2 and 4 h after CLP, did not increase bacterial burden 18 h after surgery compared to ANP-vehicle treated controls. n.s., not significant. (C) IL-1β and CXCL2 in lung protein lysate of mice 18 h after CLP. Two i.v. injections of PANP or ANP were given 2 and 4 h after CLP. Squares represent values from individual mice, and lines indicate mean values + SD *p < 0.05. (D) Nitrotyrosine formation. Photomicrographs of lung and liver sections from septicemic mice treated with ANP-vehicle or PANP. Paraffin embedded sections were stained with Ab to nitrotyrosine (red) and with DAPI to visualize nuclei (blue). Polymicrobial sepsis was induced by CLP; mice were treated with PANP or ANP 2 and 4 h after challenge and were sacrificed for tissue processing and staining 18 h after challenge. Bar measures 40 μm, lung, or 20 μm, liver. (E) Quantification of nitrotyrosine formation (ratio of nitrotyrosine^+^ cells per nucleus). Error bars indicate SD ***p < 0.001. (F) Lung wet-weight to dry weight ratio of mice instilled, i.t., with live *P. aeruginosa* (10^7^ cfu) or after CLP to induce polymicrobial sepsis or after i.p. injections of LPS. Two consecutive i.v. injections of PANP or ANP were given 2 and 4 h after challenge. Lung wet to dry weight ratio measured 6 h after challenge. Squares represent values from individual mice and lines indicate mean values. *p < 0.05. (G) Serum markers of tissue damage measured 18 h after CLP and two consecutive injections of PANP or ANP given 2 and 4 h after CLP. Lactate dehydrogenase (LDH) activity was significantly reduced in peripheral blood sera obtained from PANP-treated mice compared ANP-vehicle treated controls. Hepatocyte-specific sorbitol dehydrogenase (SDH) activity was significantly reduced by PANP treatment when compared to ANP treated controls. Squares represent values from individual mice, and lines indicate mean values + SD *p < 0.05.

Excessive ROS production is a potent mediator of tissue damage.^39,40^ We found that ANP^high^ cells were characterized by high ROS production (see Figure 6E). To test whether piceatannol administration could reduce integrin-mediated neutrophilic superoxide production,^41^ we measured ROS production by bone marrow Ly6G^+^ PMN (SI Figure 6). Bone marrow PMN responded to stimulation with the bacterial peptide fMLP with strong ROS production (SI Figure 6). PMN with higher uptake of PANP showed a greater reduction in ROS production (SI Figure 6). Moreover, the delivery of piceatannol via PANP increased drug efficacy by orders of magnitude when compared to free drug possibly because of its incorporation primarily in the toxic PMN subset (SI Figure 6). We therefore examined whether PANP treatment reduced superoxide production by the lung PMN of endotoxemic mice. We challenged mice with a lethal dose of LPS and analyzed the production of ROS by lung Ly6G^+^ PMN ex vivo. We found that ANP^high^ PMN had significantly greater intracellular ROS concentrations than ANP^low^ PMN (Figure 8A). PANP treatment drastically curtailed ROS production in ANP^high^ PMN (Figure 8A). These data demonstrated that ANP^high^ PMN were largely responsible for ROS production by lung PMN in endotoxemic mice.

Lung PMN are essential for clearing bloodstream bacteria because the resident macrophages in the liver and spleen alone are insufficient for that task.^42,43^ We therefore determined the effects of PANP treatment on the bacterial burden of mice in the CLP model of polymicrobial infection. Two consecutive i.v. injections of PANP, given 2 and 4 h after CLP, did not increase bacteremia when compared to ANP injected controls (Figure 6B). The bacterial burden of the lungs, livers, and spleens of bacteremic mice was similar in PANP-treated mice and ANP-treated controls (Figure 8B), indicating that PANP-treatment did not compromise host antimicrobial function. That PMN-dependent antimicrobial function was fully preserved after PANP treatment is consistent with our finding that ANP^low^ PMN serve as the primary antibacterial subset of PMN.

We then tested whether PANP-treatment reduced tissue inflammation by measuring the concentrations of crucial inflammatory mediators, IL-1β and CXCL2. In lung tissue extracts of mice subjected to CLP, we found substantial reductions in concentrations of IL-1β and CXCL2 after PANP treatment when compared to ANP-vehicle treated controls (Figure 8C). We also measured nitrotyrosine formation in lungs and livers of septicemic mice. Activated lung myeloid cells, as well as epithelial type II cells, can release both NO and superoxide, which react to form peroxynitrite (ONOO^–^), a potent oxidant causing tissue damage.^44^ ONOO^–^, together with NO or superoxide, is required to nitrate protein tyrosine residues.^44^ We observed that nitrotyrosine-specific staining in inflammatory and parenchymal cells was significantly reduced in lungs and livers of mice treated with PANP when compared to ANP-vehicle treated controls (Figure 8D,E).

We also determined the effects of PANP treatment on the formation of pulmonary edema, a characteristic feature of inflammatory lung injury (ALI). A marked increase in lung wet-to-dry weight ratio is indicative of breakdown of the alveolar capillary barriers, the hallmark of ALI. Pneumonia is the most common cause of ALI in patients,^45,46^ and in a model of pneumonia induced by i.t. instillation of live *P. aeruginosa* bacteria, PANP treatment significantly reduced pulmonary edema when compared to treatment with control ANP (Figure 8F). Furthermore, treatment with PANP significantly reduced pulmonary edema in endotoxemic and septicemic mice when compared to lungs from ANP-vehicle treated controls (Figure 8F). A reduction of tissue damage, because of reduced lung inflammation, could be the proximate cause of reduced ALI after treatment. Measuring a marker of overall cell damage, lactic dehydrogenase (LDH),^47^ revealed that PANP treatment significantly reduced CLP-induced activity of LDH in the serum as compared to ANP treated controls (Figure 8G). In addition, hepatocyte-specific sorbitol dehydrogenase (SDH) activity, a marker of hepatocyte damage,^48^ was also significantly reduced by PANP treatment of septicemic mice (Figure 8G).

## Conclusions

Using ANP, we established a phenotypic and functional profile of tissue-toxic ANP^high^ PMN that exist side by side with ANP^low^ PMN that were highly efficacious in bacterial killing. Furthermore, we demonstrated that ANP are efficacious drug carriers suitable to directly target tissue-toxic PMN.

Administration of ANP carrying piceatannol, a Syk inhibitor, mitigated pulmonary edema in ALI and dramatically improved survival in polymicrobial sepsis, but, critically, it did not increase the host’s bacterial burden. Syk activity in PMN has been described as essential for antibacterial function.^49^ The use of a Syk-inhibitor in the course of bacterial infection is thus only sensible when it can be restricted to the neutrophil subset that is dispensable in host antimicrobial function. An alternative explanation for the presence of ANP^low^ PMN in mice, that ANP^low^ PMN represent PMN after peracute elimination of ANP, seems unlikely to us in light of the distinct functional capabilities of ANP^low^ PMN and their prevalence in naive mice injected with ANP. ANP^low^ PMN, as we discovered, were more efficient in elimination of *E. coli* bacteria than ANP^high^ PMN. Septic patients often lose the ability to combat bacterial infection.^50^ In such patients, the overwhelming recruitment of PMN of the ANP^high^-phenotype could be partially responsible for their inability to control an infection. Increasing the number of antimicrobial ANP^low^ PMN might be beneficial in the therapy of these patients.

Changes in chemokine receptor expression have been used to delineate neutrophil subsets. Aged neutrophils, first described in vitro as functionally deficient,^51^ have subsequently been shown to promote disease in vivo in models of sickle-cell disease and endotoxin-induced septic shock.^52^ Increased expression of chemokines and chemokine receptors in ANP^high^ PMN was consistent with their role in promoting tissue inflammation.^53^ Several of the chemokines such as CCL3 and CCL4 or CXCL2 and CXCL3 are members of the macrophage inflammatory protein family and are typically thought to be released by macrophages to increase the influx of pro-inflammatory cells such as PMN.^54^ ANP^high^ PMN were characterized by higher expression of chemokine receptors such as CCR1 and CCR5 (the receptors for the ligand CCL3) and could thus promote a vicious cycle of hyper-inflammation and tissue injury.^55^ Phagocytosis is associated with the generation of ROS.^39^ ANP^high^ PMN were efficient in phagocytosis and also produced inordinate amounts of ROS. Excessive inflammation induced by ANP^high^ PMN correlated with severe tissue injury and organ failure. We showed that reducing ANP^high^ PMN inflammation via targeted PANP treatment mitigated ROS-mediated tissue injury (Figure 8). Nontargeted pharmacological inhibition of the activation of phagocyte oxidase in human PMN has been proposed as a means of suppressing oxidative damage during inflammation without blocking antimicrobial function, but lacked clinical follow-up.^56^ Programmed disarming (controlled diurnal degranulation)^57^ and depriming (the rerelease of formerly primed lung neutrophils into systemic circulation),^58^ described as a mechanism to detoxify neutrophils, may occur in vivo but would require germline manipulation to induce therapeutically.^57^ In contrast, achieving therapeutic efficacy by subset- and activation-specific targeting seem more feasible.

The in vivo properties of ANP suggested that they were distinct from those of other nanoparticle preparation, some of which are currently in clinical use, such as nanoparticle albumin bound (nab)-paclitaxel.^59^ Nab-paclitaxel preparations do not target a specifically activated leukocyte subsets but release paclitaxel into cancerous tissues.^59^ The concept of targeting neutrophils for diagnostic or therapeutic purposes has recently been further validated.^60^ Using a supramolecular arrangement of protein in or on nanoparticles, another group described a different way for targeting neutrophils.^60^ While these nanoparticle preparations showed tropism for pulmonary neutrophils, their specificity for neutrophils or whether they would target functionally distinct neutrophil subsets has not been reported.^60^ Some of these nanoparticle preparations showed intrinsic anti-inflammatory effects,^60^ potentially complicating their use as drug delivery platforms. Moreover, it has not been addressed whether these nanoparticle preparations interfere with host antimicrobial function.^60^ By contrast, our data indicate that ANP are suitable drug delivery vehicles that do not interfere with antibacterial host function. It has also been shown that incorporation of denatured albumin beads by neutrophils depends on Mac-1 expression,^61^ whereas ANP endocytosis is Mac-1-independent and requires CD16,^27^ suggesting distinct molecular mechanisms for endocytosis of albumin nanoparticles.

Given the distinct phenotypic and functional profile of ANP^high^ PMN, these cells might play a pathogenic role in COVID-19, the disease caused by coronavirus SARS-CoV-2.^62^ The main cause of COVID-19-mortality is acute respiratory failure.^63^ In patients with severe COVID-19, activated PMN, recruited to the pulmonary microvessels, produced histotoxic mediators including ROS.^64^ PMN might contribute to the cytokine release syndrome (“cytokine storm”) that characterizes severe COVID-19 disease.^65^ It is possible that therapy targeting ANP^high^ PMN in this condition might prevent a patient’s hyperinflammatory response to SARS-CoV-2 without weakening the antiviral response. Our findings may also be relevant in noninfectious settings such as cancer. The cancer microenvironment itself alters recruitment of neutrophil subsets, and this can either promote or impede tumor growth and metastasis.^12,13^ It is conceivable that one subset of PMN promotes tumor growth while another exerts growth-inhibitory effects. Altering the balance of these subsets could be exploited therapeutically.

Conceptually and evolutionarily, the separation of tumor-promoting from tumor-impeding neutrophils, of tissue-protective from tissue-destructive subsets, or of antibacterial from antiviral subsets would facilitate specific and efficient adaptation to the requirements of local microenvironments. Neutrophil-mediated pathology may thus represent a disturbed balance in neutrophil subset composition and restoring that balance, facilitated by subset-specific targeting of PMN, might be salutary in many disease conditions.

At this point we have not determined whether differential endocytosis of ANP by PMN is a function of shifts in PMN activation states or the result of persistent differences in cell differentiation and identity. Our characterization of distinct neutrophil subsets should facilitate the search for subset-specific regulators of differentiation and identity. To definitively address the question whether the capacity for endocytosis of nanoparticles (in the present case of ANP) is a distinct response of a bona fide neutrophil subset will require lineage tracing, a technique used to track other leukocyte populations that has never been applied to PMN.^66^ Our data on human neutrophils suggest that differential endocytosis of ANP reflects an evolutionary conserved trait of neutrophils, a trait useful in potential neutrophil subset-specific targeted therapy for human inflammatory diseases.

In summary, we used distinctly formulated ANP to establish a phenotypic and functional profile for a deleterious neutrophil subset. ANP facilitated a therapeutic approach that directly targets neutrophils without compromising the hosts’ protective innate immune function.

## Methods/Experimental Section

### Preparation of Albumin Nanoparticles

Bovine serum albumin (BSA, MW 66.5 kDa) was purified by acetone and a 0.2 μm filter. Glutaraldehyde (25% in water) was bought from Sigma-Aldrich. BSA concentration was measured using the Coomassie (Bradford) Protein Assay Kit (Fisher Scientific). ANP were prepared following a desolvation technique, in a modification of an earlier technique.^27^ Purified BSA was diluted to 20 mg/mL with endotoxin free water. The BSA solution (1 mL) was transformed into nanoparticles with addition of pure ethanol (3.5 mL) over 10 min while stirring at room temperature and stabilized by the addition of 38 μL glutaraldehyde left to stir for a minimum of 4 h. ANP were collected by centrifugation (15,000 g, 20 min, 4 °C) and washed three times by resuspension in endotoxin free water (1 mL). After the third wash, the pellet was resuspended in high concentration (∼20 g/mL) and stored at 4 °C prior to formulation for experiments.

### Preparation of PANP

Piceatannol (5 mg) was dissolved in DMSO (50 μL) by strong agitation, which was then added to the BSA solution (20 mg BSA, 1 mL endotoxin free water). The mixture was left stirring to incubate for a minimum of 1 h, allowing the piceatannol to interact with the solubilized BSA. The mixture remained covered in foil to prevent UV degradation of piceatannol. After 1 h, synthesis continued with the addition of ethanol and glutaraldehyde. Loading efficiency (SI Figure 7) was measured via extraction followed by LC-MS (Alliance 2795 HPLC, Quattro micro API triple quad (QQQ) mass spectrometer, Waters, Milford, MA, USA). Extraction was carried out with acetonitrile using an internal standard working solution (custom synthesized trans-Piceatannol-d3, 10 mg/mL, 20 μL) . Piceatannol (98%) was purchased from MuseChem and trans-Piceatannol-d3 was custom synthesized by Toronto Research Chemicals Inc. The endotoxin content of prepared nanoparticles was measured using a Genscript ToxinSensor Chromogenic LAL Endotoxin Assay Kit. Endotoxin content was found to be 0.109 Eu/mL for infused nanoparticle formulations at a nanoparticle concentration of 2 mg/mL. Alexa-647 (NHS ester), acetone (ACS grade), ethanol (200 proof), water for injection (WFI), endotoxin free water (HyClone), and phosphate buffered saline (PBS, 1×, without magnesium or calcium) was purchased from Fisher Scientific. Alexa-647 (25 μg) was dissolved in DMSO (10 μL). Then, the Alexa-647 solution was added to the BSA-piceatannol solution and incubated for 1 h. The synthesis then proceeded as before with the addition of ethanol. Any unloaded dye was washed out of the product during the three consecutive washes at the end of the procedure. PANP were characterized by determining size, polydispersity index (PDI), and surface charge as zeta potential (SI Figure 8). Size and PDI were measured via dynamic light scattering using a Zetasizer (ZS, Malvern Industries, Worcestershire, UK). First, the sample was diluted with 0.2 μm filtered water (1 mL). Then, a disposable cuvette was filled with 0.2 μm filtered water (1 mL). Next, seven drops of sample were added, and the cuvette was shaken lightly to mix. Finally, the cuvette was placed in the Zetasizer and measured. Zeta potential was measured by laser Doppler micro electrophoresis using a Zetasizer. The previous sample was diluted with 0.2 μm filtered water (1 mL). The sample was then added to a disposable folded capillary cell and measured. Size was verified via nanoparticle tracking analysis using a Nanosight (NS3000, Malvern Industries, Worchestershire, UK) The sample was first diluted 100,000× with 0.2 μm filtered water. Then, the sample was injected into the Nanosight via syringe pump and read at 500 nm to obtain a video and analyzed to give particle size distribution. ANP and PANP, uniform-sized spheric nanoparticles, were of consistent hydrodynamic size (120 nm ± 28 nm diameter and zeta potential (−27 ± 5.48 mV) distribution (SI Figure 7). We injected i.v. 8.3 mg/kg body weight of ANP or of ANP loaded with 8.9 μM piceatannol (PANP).

### Mice and Human Cells

Mice were treated in accordance with the NIH Guide for the Care and Use of Laboratory Animals and UIC animal care committee’s regulations. All procedures were approved by the UIC IACUC. For transcriptomic and mass cytometric analysis, we used inbred male C57BL/6J (The Jackson Laboratory) 6 to 8 wk of age because the available antibodies were mostly only tested for specific reactivity to epitopes expressed by C57Bl6 mice. For the surgical induction of polymicrobial sepsis, we used outbred male CD 1 mice male (Charles River Laboratories), at a body weight ranging from 34 to 38 g, because their genetic heterogeneity is closer to that of human populations. For adoptive transfer experiments, we used male inbred male BALB/c mice (The Jackson Laboratory) is between 8 and 10 wk old. Studies employing human peripheral blood cells obtained by venipuncture were approved by the UIC Institutional Review Board (IRB). For in vitro ANP endocytosis studies, heparinized blood was cultured at 37 °C for 4 h in the presence of LPS (100 ng/mL) or saline. AF647-labeled ANP or saline with 10% BSA was added for the last half-hour of incubation. Cells were then cooled to 4 °C and stained with specific Abs. For flow cytometric analysis, erythrocytes were lysed and cells were fixed.

### Flow Cytometry, Biodistribution, and Pharmacokinetics

Single cell suspensions were prepared as described.^67^ Cells were stained for 30 min on ice. Dead cells were excluded by F-SC, S-SC. PMN were gated by Ly6G^hi^ CD177^hi^ CD115^lo^ S-SC^hi^. Antibodies for mouse samples were from Bdbiosciences, CCR1; CXCR2; CXCR4; TCR-β (H57-597); NK-1.1 (PK136); CD16/CD32 (2.4G2); ebioscience, CD11b (M1/70), CD11c (N418), CD31 (PECAM-1, 390), CD45 (30-F11), CD64, CD115 (AFS98), F4/80 (BM8), MHC II (M5/114.15.2); R&D, CD177 (1171A); Biolegend, Ly6C (HK1.4) and Ly6G (1A8). Antibodies for human samples were from Bdbiosciences, CD66b (G10F5); CD10 (HI10 α). Isotype-matched Abs to irrelevant epitopes were used as negative controls. For organ optical imaging, fluorescence was measured by a Xenogen IVIS Spectrum (Caliper Life Sciences) and images were processed with Living Image software (ver. 4.3.1). An excitation filter of 785 nm and emission filter of 820 nm with 120 s exposure times were used for all experiments. ANP blood serum concentration was determined by measuring the fluorescence of serum samples obtained by venipuncture (retro-orbital venous sinus) at various times after administration of AF647-fluorochrome labeled ANP (SI Figure 3). ANP concentrations and standard curves, generated by serially diluting AF647-fluorochrome labeled ANP in blood serum of untreated matched control mice, were determined using a microplate reader. Peripheral blood half-life of ANP (0.38 ± 0.04 h) was calculated using one-phase decay equation under nonlinear regression in GraphPad Prism 9.1.1.

### Transcriptomic Profiling

Mice were injected i.p. with LPS (12 mg/kg) or saline; ANP were injected i.v. 1 h before mice were euthanized. PMN were harvested from lungs and by flow cytometry sorting of Ly6G^pos^ divided into ANP^high^ and ANP^low^ PMN. mRNA was isolated and prepared immediately for RNA-Seq or qPCR analysis.

#### RNA-Seq

Raw reads were aligned to reference genome mm10 using STAR.^68^ Gene expression was quantified using FeatureCounts^69^ against Ensemble coding and noncoding gene annotations. Differential expression between nanoparticle dye selection positive and negative was computed using edgeR,^70,71^ adjusting for technical batch effect due to mouse cohort selection; normalized gene expression was reported in log_2_ CPM units. *P*-values were adjusted for multiple testing using the false discovery rate (FDR) correction of Benjamini and Hochberg.^72^ Significant genes were determined based on an FDR threshold of 5% (0.05). All genes that were differentially expressed due to nanoparticle dye selection, in either LPS treated or untreated animals, were visualized in a heatmap, including dendrograms from complete linkage hierarchical clustering for both genes and samples. In addition, separate heatmaps for chemokine ligands and chemokine receptors were generated, plotting all genes with CPM > 0.25 (10 reads at a sequencing depth of 40 M reads) regardless of differential expression levels. LPS treated animals were separated from untreated animals in these heatmaps to highlight the effect of nanoparticle dye selection on the expression levels.

#### Quantitative Real-Time PCR (qPCR)

mRNA was extracted from sorted PMN using the Qiagen RNeasy Mini Kit. Total RNA quantity was measured at 260 nm, and purity was assessed by the optical density 260 nm/optical density 280 nm ratio. 0.5 μg of RNA was transcribed to complementary DNA with random primers using the High-Capacity cDNA Reverse Transcription Kit (ThermoFischer). Quantitative gene expression was evaluated by qPCR using the QuantStudio 7 Flex Real-Time PCR System. Results were calculated using the comparative C_T_-method,^73^ and expressed relative to the expression of the housekeeping gene Ppia (ENSMUSG00000071866.12). We used the following primers, forward, and reverse, respectively: Ppia GGCAAATGCTGGACCAAACAC, TTCCTGGACCCAAAACGCTC: Il1b TGGGAAACAACAGTGGTCAG, CAAGGAGGAAAACACAGGCT; Il15 CAATTCTCTGCGCCCAAAAG, TCTTAAGGACCTCACCAGC; Ccl3 AGAAGGATACAAGCAGCAGC, GACTTGGTTGCAGAGTGTCA; Ccl4 GATCTGTGCTAACCCCAGTG, AGAAGAGGGGCAGGAAATCT; Cxcl2 AGTTTGCCTTGACCCTGAAG, GTGAACTCTCAGACAGCGA; Cxcl3 GCCCCAGGCTTCAGATAATC, AAAGACACATCCAGACACCG.

### Mass Cytometry

For mass cytometry analysis, isotope labeled Abs (Fluidigm) and purified antibodies were obtained from Biolegend, ThermoFisher, R&D Systems, and conjugated using MAXPAR DN3 antibody labeling kits (Fluidigm) according to manufacturer’s instructions. Lung single cell suspensions prepared for flow cytometry sorting of CD45^+^ into ANP^high^ and ANP^low^ cells. Cells were stained with 50 mL of metal isotope-labeled surface antibodies on ice. After 30 min, cells were washed with staining buffer, once with PBS, and then fixed in 2% paraformaldehyde in PBS. Cellular DNA was labeled at room temperature with 250 nM iridium intercalator (Fluidigm) in 2% PFA/PBS. After 20 min, cells were washed twice with staining buffer. Prior to acquisition, cells were then washed once with cell staining media and then finally with water alone before running on the CyTOF. EQ Four Element Calibration Beads (Fluidigm) were added to samples prior to acquisition. Samples were acquired on a CyTOF2 (Fluidigm). After mass cytometry acquisition, data were exported in flow-cytometry (FCS) format and normalized. For unbiased clustering analysis, machine learning-driven unbiased analyses (viSNE) were performed on CyTOF data sets using Cytobank (https://www.cytobank.org/).

### Adoptive Transfer Experiments

Donor mice, male BALB/c 8 to 10 wk, were injected with one i.p. dose of LPS [30 mg/kg]. Prior to euthanasia, mice were injected, into the tail vein, with ANP containing the fluorochrome AF647. After euthanasia, both heart and lungs were perfused with PBS, excised lung lobes were minced and digested in collagenase solution. Erythrocytes were lysed. Syngeneic recipient mice of the same age were injected, i.p., with a nonlethal dose LPS [1 mg/kg] prior to adaptive transfer, i.v., of 8 × 10^5^ ANP^high^ or an equal number of ANP^low^ granulocytes.

### CLP and Induction of Lung Injury

Mice received a single dose of LPS (*Escherichia coli* 0111:B4, InvivoGen) intraperitoneally. Polymicrobial sepsis was induced by cecal ligation and puncture using a 16-gauge needle. In sham controls, only laparotomy plus cecal ligation without puncture of the cecum was performed.^35^ For survival studies, mice were monitored twice daily for 6 d. i.t. instillation of live *P. aeruginosa* bacteria was performed as described and used to induce ALI.^74^

### Determination of Bacterial Load

We collected samples 4 h after CLP. The blood and digested tissue samples were suspended in sterile distilled water, and dilutions (1:2, 1:100, 1:1,000) were prepared and plated on LB agar plates. Plates were incubated for 24 h, bacterial colonies were counted, and the number of colony-forming units per mL blood was calculated.^67^

### Phagocytic and Intracellular Killing

Phagocytosis and bacterial killing were determined by adapting a previously described method.^75^ Briefly, the deterioration of GFP was monitored in Ly6G^+^ PMN by flow cytometry between 1 and 4 h after exposure of Ly6G^+^ PMN to GFP-expressing *E. coli*. Killing was defined as the percentage of fluorescence (*E. coli-*GFP), detected 1 h post incubation of PMN with *E. coli*-GFP, remaining 4 h post incubation and 3 h post removal of nonphagocytosed bacteria from the neutrophil-incubation medium. Average (*n* = 3) of fluorescence detected at 1 h = 100%; % killing = 100 – percentage of fluorescence detected at 4 h post start of incubation.

### Tissue Damage Markers

The activity, LDH and SDH, was determined using commercial kits according to manufacturers’ instructions. Histopathology was evaluated in sections from paraffin embedded or frozen tissues using specific antibodies to nitrotyrosine as described.^76^

### Quantification of Hydrogen Peroxide Production

We measured hydrogen peroxide production using the Amplex Red Hydrogen Peroxide Kit (Invitrogen) following the manufacturer’s instructions. For PMN, some were flow cytometry sorted according to their endocytosis, and ANP were washed and resuspended in HBSS buffer plus 1% glucose. We incubated 2 × 10^4^ cells with Amplex Red reaction mixture with 10^–7^ M of fMLP or Phorbol ester (PMA (2 ng/mL) at 37 °C for 5 min prior to measurements with a fluorimetric plate reader at an excitation wavelength of 544 nm and an emission wavelength of 590 nm, or absorbance at 560 nm was measured. For ex vivo measurement of ROS production we used dihydrorhodamine 123.^77,78^ Male CD1 mice were injected with one i.p. dose of LPS [40 mg/kg]; 1 and 2 h later, mice were injected with fluorochrome labeled ANP or PANP as described above; 6 h after LPS challenge, mice were euthanized and heart and lungs were perfused with PBS; excised lung lobes were minced and digested in collagenase solution. Erythrocytes were lysed. Leukocytes were enriched by Ficoll density gradient. Cells were resuspended in HBSS plus 1% glucose incubated with dihydrorhodamine 123 for 20 min at 37 °C and then immediately processed and analyzed by flow cytometry.

### *In Vivo* Imaging

Two-photon microscopy was performed as described.^38^ Briefly, surgical methods to access to the lung are based on Looney et al.^79^ Tail vein injection with BV421-labeled Ly6G antibody (10 μg/mouse) (1A8, Biolegend) and 70 kDa tetramethylrhodamine-dextran (200 μg/mice) (ThermoFisher Scientific) were performed to stain PMN and lung microvascular structures, respectively, before surgery. A resonant-scanning two photon microscope (Ultima Multiphoton Microscopes, Bruker) with an Olympus XLUMPlanFL N 20x (NA 1.00) collected dual-color images (Emission filter; 460/50 nm for Brilliant Violet 421 and 595/60 nm for tetramethylrhodamine) with 820 nm excitation at video rate. Images were processed and analyzed by Image J and customized LabVIEW programs. For PMN amount analysis, fluorescent intensities of PMN in the field of view were quantified, and the value of saline injected controls was normalized to 1. For PMN internalizing P/ANP, PMN number with or without P/ANP in the field of view was counted and the percentage was calculated. For PMN velocity analysis, PMN velocity of cells migrating more than 1 min in the field of view was measured.

### Statistical Analysis

We examined the differences between groups for statistical significance by Student’s *t* test or ANOVA and compared survival curves with a log-rank test. Enrichment of chemokine receptor or chemokine expression in the ANP^high^ and ANP^low^ groups was assessed by Fisher’s Exact Test. A *p* value of <0.05 was considered statistically significant.

## Supporting Information Available

Eight Supplemental Figures, illustrating the characterization, biodistribution, and pharmacokinetics of the nanoparticles (PDF)
Two-photon microscopy video (MP4)
Supplemental data (XLSX)
