# Supplementary material for: Albumin Nanoparticle Endocytosing Subset of Neutrophils for Precision Therapeutic Targeting of Inflammatory Tissue Injury
Source: ACS Nano. 2022 Mar 1;16(3):4084–101. doi: 10.1021/acsnano.1c09762 (PMC8945372; doi:10.1021/acsnano.1c09762)
Supplement: Supplementary file 1 — nn1c09762_si_001.pdf [file nn1c09762_si_001.pdf]

SUPPORTING INFORMATION

Albumin Nanoparticle Endocytosing Subset of Neutrophils for Precision Therapeutic Targeting of Inflammatory Tissue Injury

Kurt Bachmaier[1,2*], Andrew Stuart[2], Abhalaxmi Singh[1,2], Amitabha Mukhopadhyay[1], Sreeparna Chakraborty[1], Zhigang Hong[1], Li Wang[1,4], Yoshikazu Tsukasaki[1], Mark Maienschein-Cline[3], Balaji B. Ganesh[3], Prasad Kanteti[2], Jalees Rehman[1,4], Asrar B. Malik[1,2 *]

[1]Department of Pharmacology and Regenerative Medicine and the Center for Lung and Vascular Biology, The University of Illinois College of Medicine, E403, 835 S. Wolcott Avenue, Chicago, IL 60612, USA

[2]Nano Biotherapeutics, Inc., 2201 W Campbell Park Dr, Chicago, IL 60612, USA

[3]Research Resources Center, University of Illinois at Chicago, Chicago, IL 60612, USA

[4]Division of Cardiology, Department of Medicine, The University of Illinois College of Medicine, Chicago, IL 60612, USA

*Corresponding Authors

Two-photon microscopy video

Two-photon microscopy of perfused, ventilated lungs from endotoxemic mice and saline-injected controls. mice were injected with LPS [30 mg/kg], 6h prior to, and with ANP or PANP 3h and 4h prior to *in vivo* imaging. Dextran traces blood vessels, outlining lung microvascular structures, blue; fluorescent-labeled Ly6G Abs label PMN, green; fluorochrome AF647 labels ANP or PANP, red.

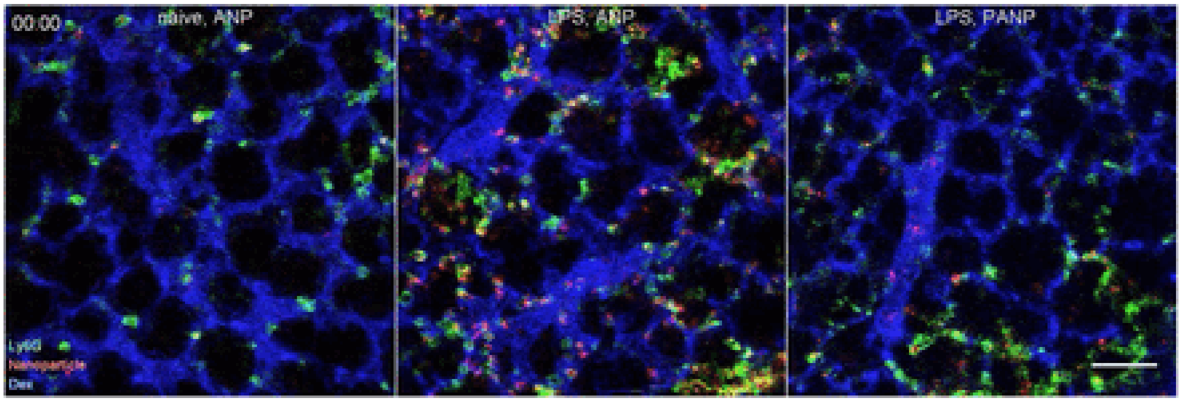

Colors are pseudo-colors. Bar measures 50µm. Representative data from a minimum or 4 mice per treatment group.

Supplemental Figures

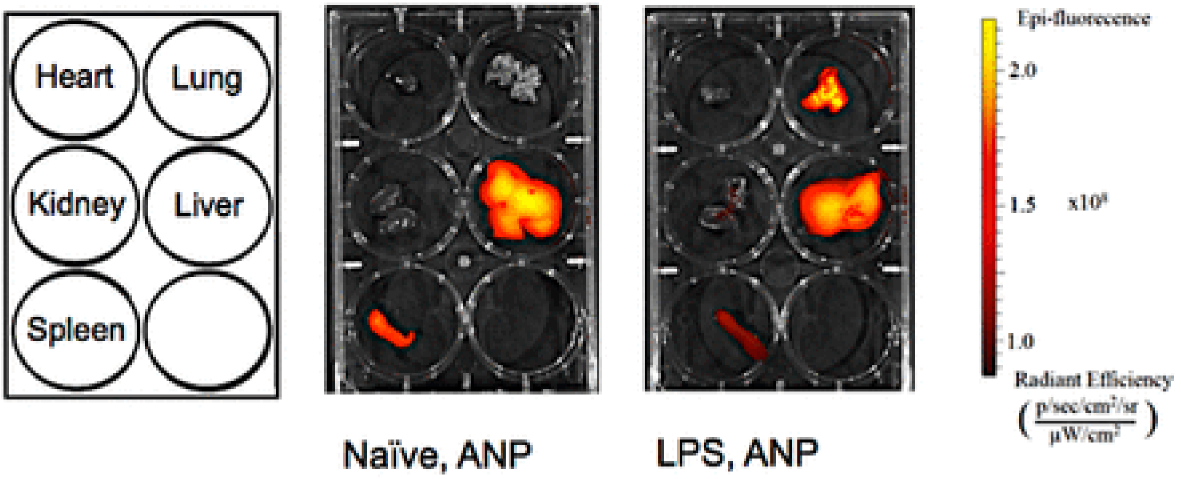

Supplemental Figure 1. ANP biodistribution measured by IVIS. In naïve mice, *i.v.*-injected ANP were mostly found to min and spleen. After *i.p.* administration of LPS [30 mg/kg], ANP appeared prominently in lungs, and remained visible in liver and in spleen. Albumin was labeled with vivotag 800 (PerkinElmer) and then formed into ANP. Organs were harvested 4 h after 150μl tail vein injection of 2 mg/mL vivotag 800 labeled ANP. Epi-fluorescence radiance scale corresponding to images. Representative data obtained from at least 3 mice per treatment group.

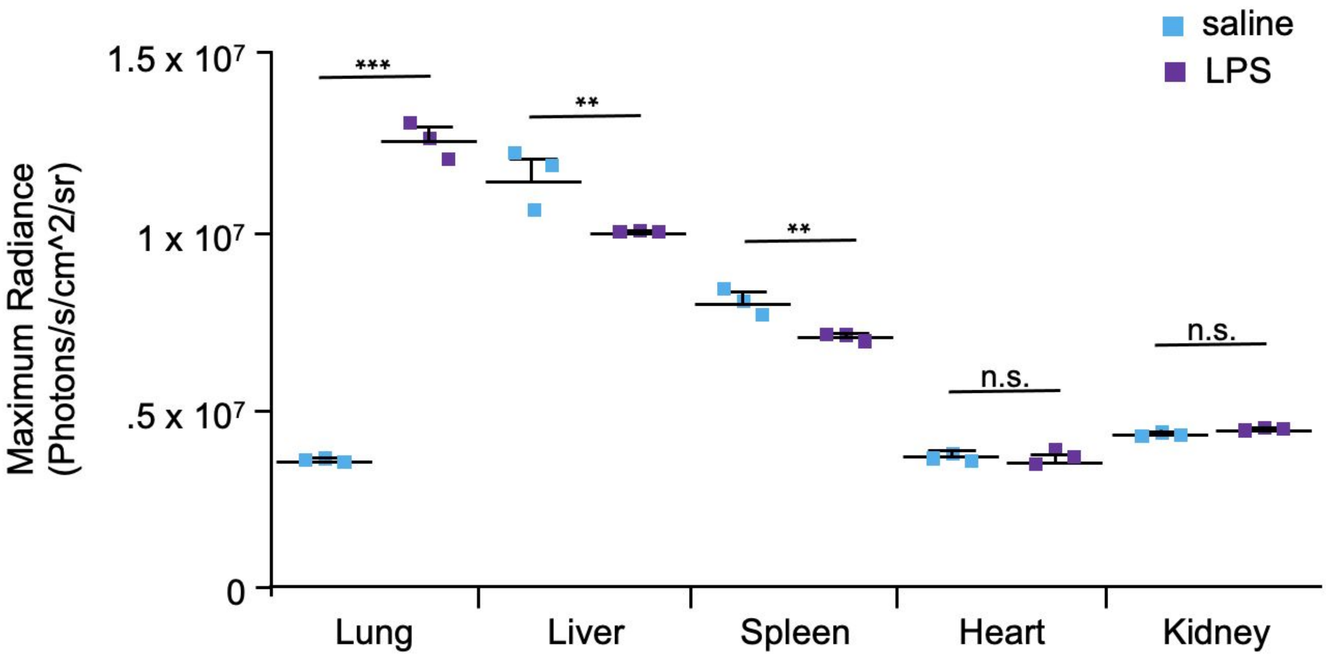

Supplemental Figure 2. Quantification of maximum radiance in organs (*ex vivo*) 4h after administration of AF647-ANP to naïve (saline) or LPS-challenged mice by IVIS. Radiance analysis was performed using Aura image analysis software. Squares represent radiance measured in organs of individual mice, lines indicate mean values + SD. $^{**}p < 0.002$, $^{***}p < 0.0001$

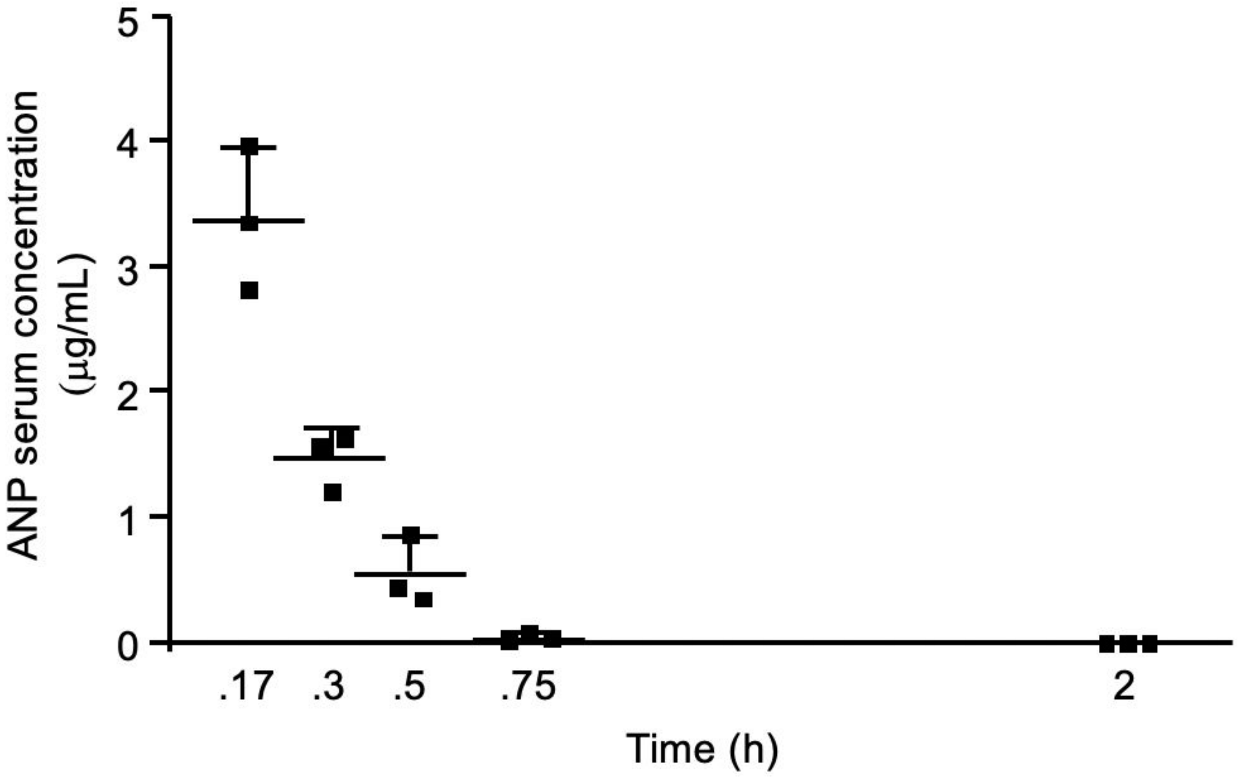

Supplemental Figure 3. Pharmacokinetics of ANP in blood serum. Squares represent ANP concentrations measured at the indicated times after ANP administration in blood sera. Blood serum half-life was 0.38 ± 0.04 h. Half-life was calculated using One-phase decay equation under non-linear regression in GraphPad Prism 9.1.1. Squares represent values from individual mice, lines indicate mean values + SD.

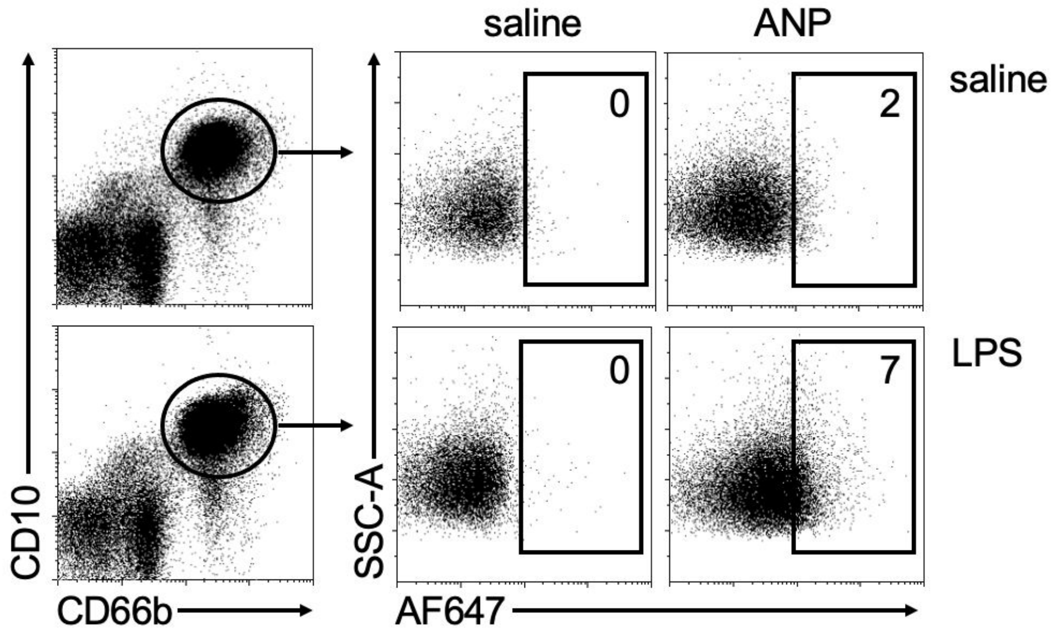

Supplemental Figure 4. Duality of ANP endocytosis by human peripheral blood PMN. (A) Flow cytometric analysis of single cell suspensions of human peripheral blood. PMN were defined as $CD10^{+}CD66b^{+}$ cells. A minority of PMN was found to endocytose ANP. Stimulation with LPS (100ng/mL) increased the percentage of PMN that endocytosed ANP from 2% to 7%. Cells were cultured at 37ºC for 4h. Incubation with ANP was for the last half hour only. Data representative for three individual donors are shown.

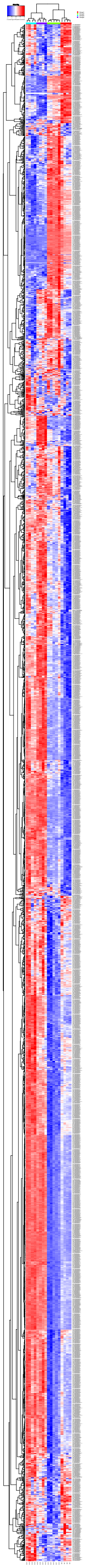

Supplemental Figure 5. High resolution representation of Figure 2B

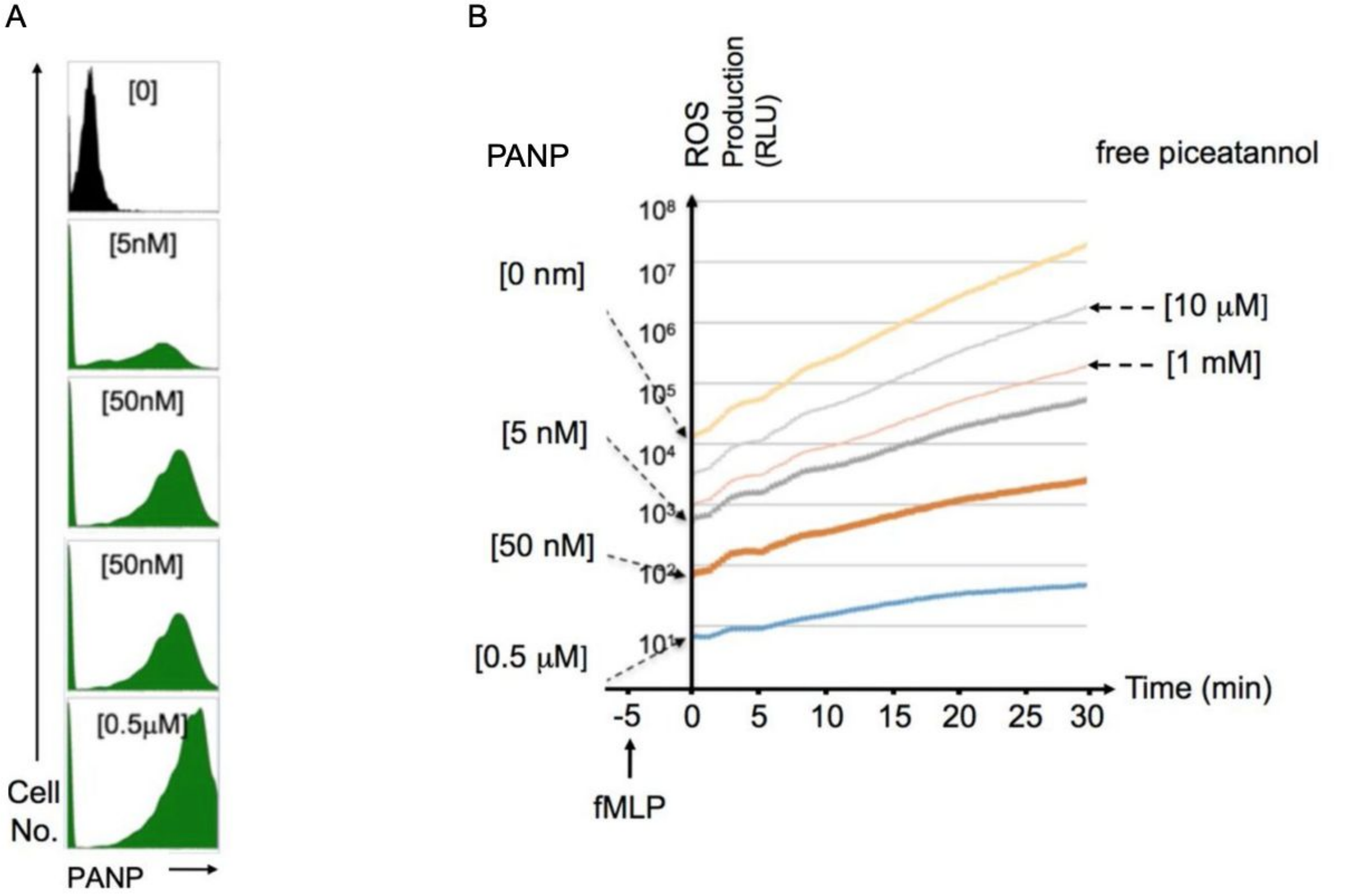

Supplemental Figure 6. PANP treatment reduces PMN superoxide production in response to fMLP stimulation. (**A**) Endocytosis of PANP. Flow cytometry histograms of bone marrow cells stimulated with LPS [1 mg/mL] and incubated with PANP, at the indicated doses, for 30min at 37ºC, and then processed for flow cytometry. Representative data obtained from at least 3 mice per treatment group. (**B**) PAMP reduced fMLP stimulated ROS production in a dose- dependent manner. Bone marrow PMN were incubated with the indicated doses of PANP and stimulated with fMLP for the indicated time. Representative data obtained from at least 3 mice per treatment group.

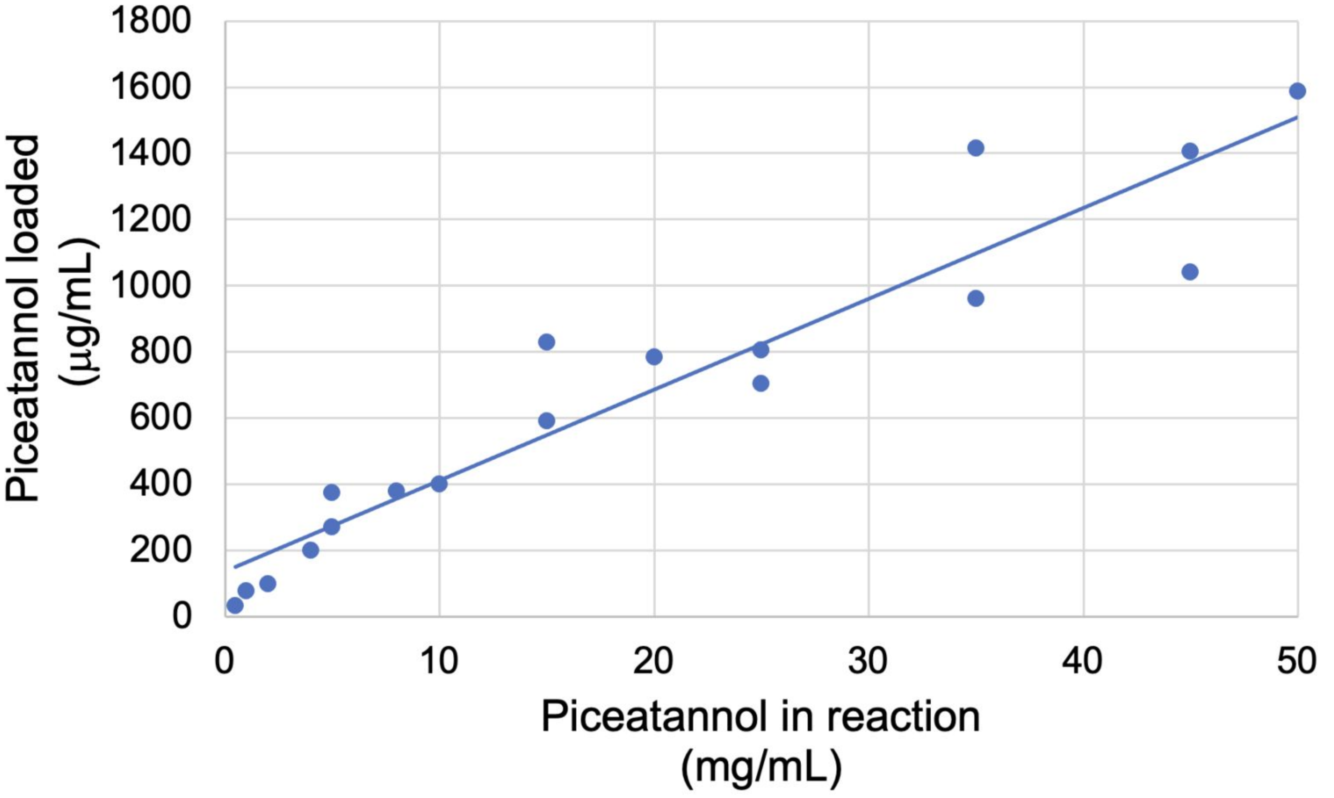

Supplemental Figure 7. Piceatannol loading efficiency of PANP preparations. Piceatannol loading experiments at various concentrations were carried out and loaded piceatannol concentrations were measured with LC-MS as described in the methods section.

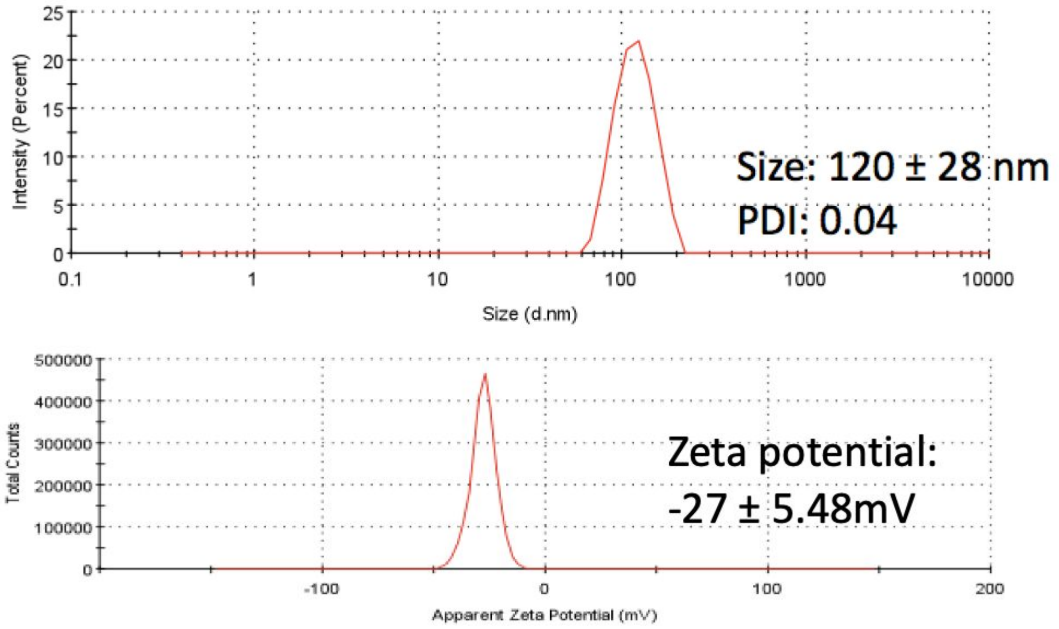

Supplemental Figure 8. Nanoparticle size distribution and surface charge. Results are representative of both ANP and PANP preparations. Polydispersity index (PDI), surface charge as zeta potential.
